# Task-specific cortical mechanisms of taVNS-paired task-oriented training for post-stroke upper extremity dysfunction under cognitive load: an fNIRS study

**DOI:** 10.3389/fnhum.2025.1652612

**Published:** 2025-09-24

**Authors:** Shi-Yi Li, Ke Xu, Yi-Xiu Wang, Meng-Huan Wang, Shu-Shan Li, Feng Lin, Zhong-Li Jiang

**Affiliations:** ^1^School of Rehabilitation Medicine, Nanjing Medical University, Nanjing, Jiangsu, China; ^2^Department of Rehabilitation Medicine, The First Affiliated Hospital of Nanjing Medical University, Nanjing, Jiangsu, China; ^3^Department of Rehabilitation Medicine, West China Hospital, Sichuan University, Chengdu, China; ^4^School of Chinese Language and Culture, Nanjing Normal University, Nanjing, Jiangsu, China; ^5^Department of Rehabilitation Medicine, Sir Run Run Hospital, Nanjing Medical University, Nanjing, Jiangsu, China

**Keywords:** transcutaneous auricular vagus nerve stimulation, task-oriented training, functional near-infrared spectroscopy, motor-evoked potentials, neuroplasticity, upper extremity rehabilitation

## Abstract

**Objective:**

This study aimed to investigate the cortical task-specific response patterns underlying the improvement of upper limb dysfunction in stroke patients using transcutaneous auricular vagus nerve stimulation (taVNS) paired with task-oriented training (TOT) under varying cognitive loads.

**Methods:**

In this randomized, double-blinded, sham-controlled trial, 30 patients with subacute stroke were enrolled and randomly assigned to either the taVNS group or the Sham group. Both groups received 3 weeks of TOT. The taVNS group received concurrent active taVNS, while the Sham group received concurrent sham stimulation. Assessments were performed pre- and post-intervention. Clinical function was evaluated using the Fugl-Meyer Assessment-Upper Extremity (FMA-UE), Montreal Cognitive Assessment (MoCA), Fatigue Severity Scale (FSS), and Modified Barthel Index (MBI). Neurophysiological measures included heart rate variability (HRV) to assess taVNS efficacy and motor-evoked potentials (MEPs) to assess cortical excitability changes. Brain functional imaging was conducted using functional near-infrared spectroscopy (fNIRS) during motor tasks with different cognitive loads (low-load: continuous horizontal movement; high-load: goal-directed movement) to analyze changes in spontaneous neural activity, task-related regional brain activation characteristics, and brain functional network alterations.

**Results:**

(1) Post-intervention, the taVNS group showed significantly greater improvements in all HRV indices compared to the Sham group (*P* < 0.05). (2) Both groups exhibited significant improvements from baseline in FMA-UE, MoCA, MBI, and FSS scores (*P* < 0.05), with the taVNS group demonstrating significantly greater improvement than the Sham group (*P* < 0.05). (3) MEP results indicated significant improvements in the elicitation rate of ipsilesional MEPs within the taVNS group post-intervention (*P* < 0.05). Furthermore, compared to the Sham group, the taVNS group showed significantly greater improvements in the ipsilesional MEP elicitation rate and a significant reduction in contralesional MEP latency (*P* < 0.05). (4) Regarding resting-state fNIRS, the taVNS group exhibited higher Amplitude of Low-Frequency Fluctuation (ALFF) values post-intervention in the ipsilesional prefrontal cortex (PFC), dorsolateral prefrontal cortex (DLPFC), and sensorimotor cortex (SMC) compared to the Sham group (*P* < 0.05), but these differences were not significant after correction. In task-state fNIR under the low-cognitive-load condition, activation levels in the ipsilesionalS primary motor cortex (M1) and premotor and supplementary motor areas (pSMA) were significantly higher in the taVNS group compared to the Sham group post-intervention (*P*_FDR_ < 0.05). During the high-cognitive-load task, activation levels in the ipsilesional PFC and DLPFC were significantly higher in the taVNS group compared to the Sham group post-intervention (*P*_FDR_ < 0.05). (5) Functional network analysis using complex network metrics revealed that the taVNS group exhibited significantly increased nodal clustering coefficient and nodal local efficiency in the ipsilesional DLPFC during the high-cognitive-load task post-intervention compared to the Sham group (*P*_FDR_ < 0.05).

**Conclusion:**

taVNS paired with TOT enhances autonomic homeostasis, increases corticospinal pathway excitability, activates cognition-motor related brain regions, and modulates functional connectivity networks through multi-pathway neuroregulatory mechanisms. This promotes the formation of task-specific cortical activation and network connectivity during motor tasks under varying cognitive demands in stroke patients. These changes contribute to improved executive control performance in complex tasks, thereby enhancing cognitive-motor integration capabilities and facilitating upper limb functional recovery.

**Clinical Trial Registration:**

https://www.chictr.org.cn/index.html, Unique Identifier/Registration Number: ChiCTR2400085163.

## 1 Introduction

Stroke has emerged as the leading cause of neurological disability worldwide. Recent epidemiological data indicate a rising annual incidence rate, with approximately 70% of survivors experiencing persistent upper limb motor dysfunction (GBD 2021 Diabetes Collaborators, 2023). Such post-stroke deficits critically compromise activities of daily living, diminish social participation, and significantly impair quality of life (De Iaco et al., 2024). Patients commonly exhibit significant fine motor deficits and intentional motor impairments during the subacute and chronic stages. Conventional rehabilitation approaches demonstrate substantial limitations in improving upper limb function (Kwakkel et al., 2019). In recent years, transcutaneous auricular vagus nerve stimulation (taVNS) has gained considerable research interest as a novel non-invasive neuromodulatory intervention, principally attributable to its unique biphasic neuromodulatory properties. By stimulating vagal afferent fibers within the auricular concha, taVNS activates the nucleus tractus solitarius and locus coeruleus, thereby upregulating the release of cholinergic (Horinouchi et al., 2024) and noradrenergic neurotransmitters (Szeska et al., 2025). This cascade ultimately promotes long-term potentiation within the motor cortex (Steidel et al., 2021). Preclinical evidence has confirmed that taVNS paired with motor training increases synaptic density in the motor cortex by 37% and facilitates the remodeling of the ipsilateral corticospinal tract in stroke models (Meyers et al., 2018). Clinical investigations further demonstrate that adjunctive taVNS significantly enhances Fugl-Meyer Assessment-Upper Extremity (FMA-UE) scores in chronic stroke patients compared to training alone (Lin et al., 2024). Nevertheless, current research predominantly focuses on behavioral improvements, lacking systematic elucidation regarding the temporal dynamics of its cortical effects (de Melo et al., 2023). Our prior work has provided preliminary evidence supporting the beneficial effects of taVNS on upper limb motor recovery in stroke patients and revealed its neuromodulatory potential on cortical activation patterns (Wang et al., 2024b). Building upon this foundation, rigorous investigation of taVNS-modulated, task-specific cortical dynamics is warranted. Existing studies suggest that cognitive engagement critically modulates functional activation characteristics within key regions, including the motor cortex (M1) and prefrontal cortex (PFC) (Meulenberg et al., 2023; Wang et al., 2023). Consequently, identifying differences in cortical responses to taVNS under varying cognitive loads is essential for a deeper understanding of cognitive-motor interaction mechanisms in stroke rehabilitation. Based on this background, this study introduces motor tasks under graded cognitive load conditions. We utilize functional near-infrared spectroscopy (fNIRS) to observe taVNS-induced, task-specific cortical activation patterns. As an emerging non-invasive neuroimaging modality, fNIRS provides high temporal resolution and motion compatibility, enabling real-time capture of oxyhemoglobin (HbO) dynamics during task execution. This makes it an effective tool for investigating taVNS-induced cortical neuroplasticity (An et al., 2025). Employing a randomized controlled design, hemiplegic stroke patients will receive combined taVNS and task-oriented training (TOT). By integrating measures including heart rate variability (HRV), fNIRS, and motor-evoked potentials (MEPs), we aim to explore the neuromodulatory mechanisms of taVNS-paired TOT on cortical excitability and autonomic function in stroke patients. Furthermore, we will discuss the specific cortical activation patterns elicited by motor tasks under different cognitive loads. This investigation seeks to elucidate the critical role of cognitive-motor interactions in the neuroplasticity facilitated by taVNS, thereby establishing a theoretical foundation for the rehabilitation of post-stroke limb dysfunction.

## 2 Materials and methods

### 2.1 Participants

This trial was conducted in the Department of Rehabilitation Medicine at Sir Run Run Hospital of Nanjing Medical University between June 2024 and March 2025. A total of 30 stroke patients were enrolled. The inclusion criteria were as follows: (1) aged 18–80 years; (2) first-ever unilateral stroke confirmed by computed tomography (CT) or magnetic resonance imaging (MRI), with a disease duration of 1–6 months; (3) Fugl-Meyer Assessment-Upper Extremity (FMA-UE) score of 20–50 on the affected side; (4) Montreal Cognitive Assessment (MoCA) score ≥18, indicating the ability to cooperate with assessments and interventions; (5) provision of written informed consent. Exclusion criteria included: (1) implanted electronic devices, intracranial vascular clips, or other electrically sensitive medical devices; (2) compromised skin integrity in the stimulation area; (3) severe end-stage cardiovascular, pulmonary, or other systemic diseases; (4) a history of vagus nerve injury; (5) upper limb dysfunction not attributable to stroke; (6) use of neuroactive medications within the past 3 months; and (7) resting heart rate < 60 beats per min. The trial was conducted following the principles outlined in the Declaration of Helsinki and was approved by the Ethics Committee of Sir Run Run Hospital, Nanjing Medical University (No. 2024-SR-034). The trial was registered at the Chinese Clinical Trial Registry (ChiCTR2400085163).

### 2.2 Sample size estimation

Sample size estimation was performed using analysis of covariance (ANCOVA) in G^*^Power 3.1.9.7. The effect size was derived from the partial eta squared value (partial η^2^ = 0.3362) for FMA-UE, as reported in the study by Wang et al. (2024b). Assuming a statistical power (1–β) of 80% and a two-tailed significance level of α = 0.05, and accounting for an anticipated dropout rate of 20%, the required sample size was calculated to be at least 12 participants per group, resulting in a minimum total of 24 participants.

### 2.3 Study design

**Figure 1:**
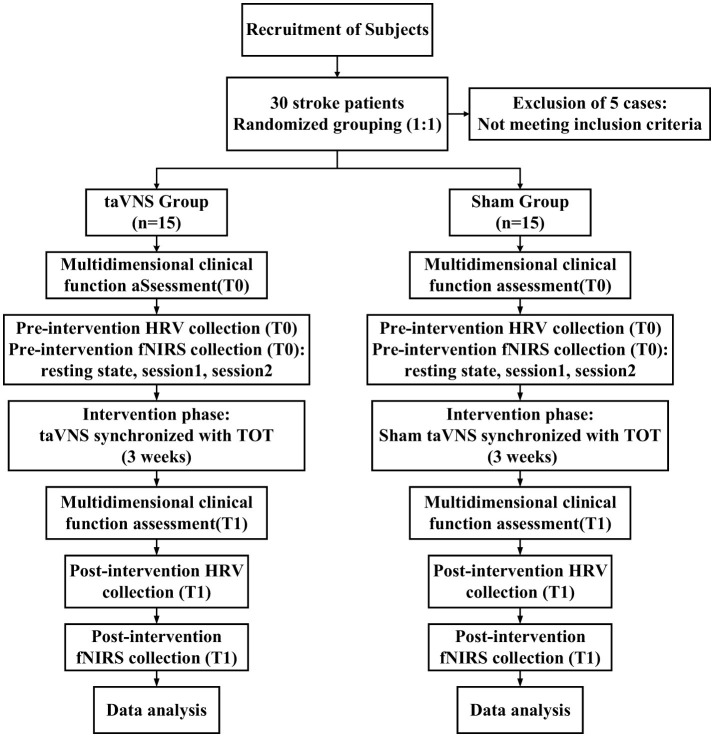
Study flow diagram. taVNS, transcutaneous auricular vagus nerve stimulation; HRV, heart rate variability; fNIRS, functional near-infrared spectroscopy; TOT, task-oriented training.

This study adopted a randomized, double-blinded, sham-controlled trial design. A total of 35 patients with subacute stroke were initially recruited, of whom 5 were excluded for not meeting the inclusion criteria. The remaining 30 eligible participants were randomly assigned in a 1:1 ratio to either the intervention group (taVNS group) or the sham stimulation group (Sham group) using a random number table. Participants in the taVNS group received taVNS combined with TOT, while those in the Sham group underwent TOT with sham stimulation. The intervention lasted for 3 weeks, with sessions conducted 5 days per week, 1 h per day. Assessments were performed at baseline and post-intervention by trained therapists blinded to group allocation. A double-blind protocol was strictly followed: participants, outcome assessors, and data analysts remained unaware of group assignments, while only the research personnel administering the intervention had access to allocation information. All interventions were administered following a standardized protocol and schedule to ensure methodological rigor and the reliability of outcomes. No intervention-related adverse events were reported by any of the enrolled participants. The study flow is illustrated in Figure 1.

### 2.4 Intervention protocol

#### 2.4.1 Task-oriented training (TOT)

Both groups received a standardized TOT protocol, supervised or assisted by licensed occupational therapists. Each training session lasted for 1 h per day, 5 days per week, over 3 weeks. The training protocol was developed in accordance with evidence-based rehabilitation guidelines (Billinger et al., 2014), and included six structured tasks: (1) forearm supported on an adjustable-height table; (2) finger-to-nose pointing exercises; (3) wrist extension to touch a target with the elbow flexed at 90 °; (4) grasping and holding a 500 mL water bottle while maintaining the same posture; (5) transferring peanuts from a cup to a plate with the affected hand, minimizing compensatory trunk movements; (6) mirror therapy using the Gloreha Professional 2 hand rehabilitation robot (Idrogenet, Italy). During each session, therapists dynamically adjusted task parameters including movement speed, distance, and resistance based on the patient's motor ability and rehabilitation goals. Visual and tactile cues were provided to facilitate accurate execution of each movement.

#### 2.4.2 Transcutaneous auricular vagus nerve stimulation (taVNS)

taVNS was administered using the Auricular Vagus Nerve Stimulator (tVNS 501, RISHENA Co., Ltd., Changzhou, China). Participants in the taVNS group received active taVNS simultaneously during each TOT session. The stimulation was delivered via a dedicated ear-clip device equipped with two dot-like electrodes, which were applied to the left cymba conchae following routine antiseptic cleansing. Stimulation parameters according to the international consensus for minimum reporting standards (Farmer et al., 2021): biphasic square pulses with a pulse width of 500μs, frequency of 25 Hz, with 30 s of stimulation alternating with 30 s of rest (duty cycle 1:1). The current intensity was individually adjusted to a comfortable level, defined as clearly above the sensory threshold but below the pain threshold. For each participant, stimulation was gradually increased from 0 mA until a distinct but non-painful tingling sensation was reported at the stimulation site (Wang et al., 2024b). The final intensity was set at the maximum level that could be tolerated without discomfort or pain, within a range of 1–10 mA (mean intensity in the taVNS group: 5.27 ± 0.98 mA). Participants in the Sham group wore an identical ear-clip device applied to the left cymba conchae and underwent the same stimulation threshold calibration procedure to maintain procedural consistency. However, during the intervention, no actual current was delivered. The electrodes were non-functional, and the stimulator displayed simulated current values and auditory signals to mimic active stimulation. Although no formal blinding assessment was conducted, no participants reported suspicion about their treatment allocation. This sham protocol has been previously validated in taVNS studies to maintain effective blinding (Wang et al., 2024b). The duration and frequency of stimulation were identical between the two groups (60 min per session, concurrent with TOT), ensuring comparability of intervention conditions across groups.

### 2.5 Outcome measures

#### 2.5.1 Multidimensional clinical function assessment

This study employed a series of standardized clinical scales to quantitatively assess improvements in upper limb motor function, cognitive ability, fatigue, and activities of daily living among participants.

Upper extremity function assessment: The Fugl-Meyer Assessment-Upper Extremity (FMA-UE) was used, comprising 33 items with a maximum score of 66. This scale is widely validated and commonly applied in the evaluation of motor impairment following stroke, with higher scores indicating better upper limb motor function (Ase et al., 2025; Wang et al., 2024c).

Cognitive function assessment: The Montreal Cognitive Assessment (MoCA) was utilized, with a total score of 30, covering multiple cognitive domains including attention, memory, language, executive function, and visuospatial abilities. MoCA is frequently employed to evaluate multidimensional changes in cognitive function and serves as an important indicator of cognitive rehabilitation outcomes (Wei et al., 2022).

Fatigue assessment: The Fatigue Severity Scale (FSS) was used to measure participants' subjective experience of fatigue. The FSS consists of 9 items, each rated on a 7-point scale (1–7), with higher total scores indicating more severe fatigue. The FSS is sensitive to changes in fatigue levels throughout the rehabilitation process (Almhdawi et al., 2021).

Activities of daily living assessment: The Modified Barthel Index (MBI) was used to assess participants' basic functional independence in daily activities. The MBI includes 10 items (e.g., feeding, dressing, toileting), with a total score of 100. Higher scores indicate greater independence and are considered a core indicator of improvement in daily functional capacity (Li et al., 2021a; Pignolo et al., 2022).

All clinical assessments were conducted at two time points, baseline (T0) and post-intervention (T1), by professional evaluators with standardized training, and changes in scores from T0 to T1 were compared to evaluate the effects of the intervention.

#### 2.5.2 Heart rate variability (HRV) assessment

HRV reflects the variation in time intervals between successive heartbeats and is widely recognized as a biomarker of vagal nerve activity (Laborde et al., 2017). In this study, resting-state HRV was continuously collected for 5 min prior to fNIRS acquisition using an SA-3000P electrocardiograph (Medocore, South Korea) under quiet conditions. The time-domain HRV metrics extracted included the average heart rate (HR), the standard deviation of the normal-to-normal (NN) intervals (SDNN), and the square root of the mean squared differences of successive NN intervals (RMSSD). For frequency-domain analysis, the ratio of low-frequency to high-frequency power (LF/HF ratio) was recorded. Previous research (Rodrigues et al., 2024; Machado et al., 2022) has demonstrated that enhanced vagal activity is typically associated with increased SDNN and RMSSD, along with decreased HR and LF/HF ratio. Accordingly, these HRV indices may serve as potential indicators of taVNS-induced modulation of vagal efferent function (Wang et al., 2024a). HRV data were collected at two time points-baseline (T0) and post-intervention (T1), to evaluate the efficacy of taVNS.

#### 2.5.3 Motor-evoked potentials (MEPs) assessment

Motor-evoked potentials (MEPs) refer to electromyographic responses recorded from target muscles following single-pulse transcranial magnetic stimulation (TMS) applied to the primary motor cortex (M1). MEPs are commonly used to assess cortical excitability and the integrity of the corticospinal tract (Paparella et al., 2020). In this study, single-pulse TMS was delivered to the hand representation areas of both the lesioned and non-lesioned M1 using a figure-eight coil (YRD CCY-I, Wuhan Yiruide). Surface electromyography (sEMG) electrodes were placed on the bilateral first dorsal interosseous (FDI) muscles to serve as recording sites (Paparella et al., 2020). The sEMG settings were as follows: sampling rate of 5,000 Hz, amplification × 500, notch filter at 50 Hz, and low-pass filter at 500 Hz. The initial stimulation intensity was set at 30% of the maximum stimulator output (MSO) (Gardi et al., 2024), and gradually increased in 5% increments until MEPs were elicited in at least 5 out of 10 consecutive trials, with peak-to-peak amplitudes ≥50 μV. The primary outcome measures included the average MEP latency and peak-to-peak amplitude recorded from bilateral FDIs. Latency was defined as the time interval between the TMS pulse and the onset of the MEP in the target muscle, while amplitude referred to the voltage difference between the MEP peak and trough. Post-intervention MEPs were elicited using the same stimulation intensity as at baseline. If no valid MEPs were detected even at 100% MSO, the result was recorded as “Not Elicited” (NA).

#### 2.5.4 Functional near-infrared spectroscopy (fNIRS) assessment

**Figure 2:**
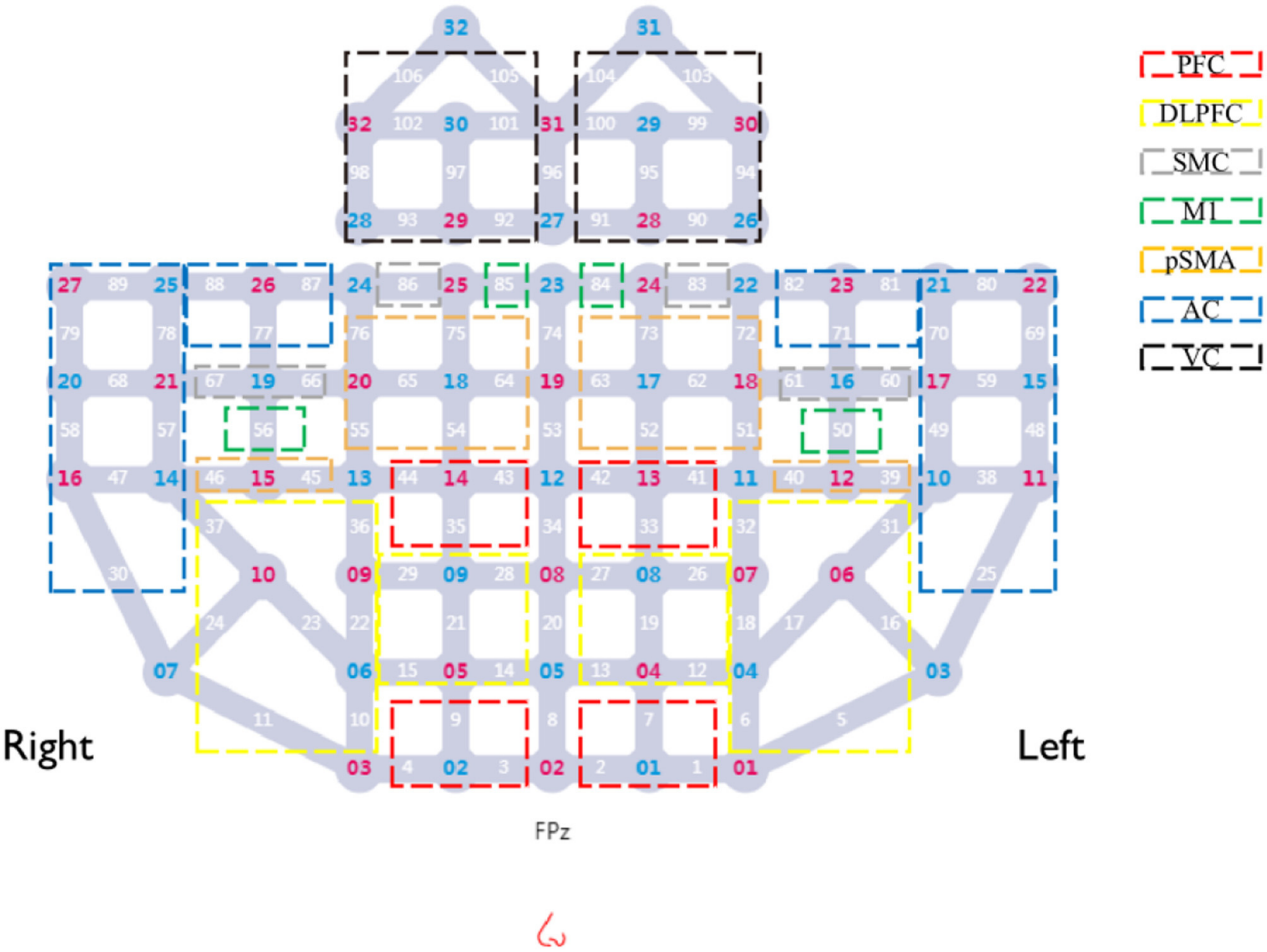
Layout of fNIRS optodes and channels. Red numbers, sources; blue numbers, detectors; white numbers, channels; colored boxes, each colored box represents a distinct Region of Interest (ROI). Channels enclosed within a box correspond to the respective ROI.

Cerebral hemodynamic signals were acquired using a multichannel continuous-wave fNIRS system (BS-3000, Wuhan ZiLian HongKang, China). The system comprises 32 semiconductor laser sources (λ1|2 = 690|830 nm, average power ≥30 mW) and 32 avalanche photodiode detectors, with a sampling frequency of 20–100 Hz. The sources and detectors were arranged over the frontal, parietal, temporal, and occipital cortices according to the 10–20 international standard electrode placement system, establishing 106 channels. A 3D spatial digitizer was used to mark anatomical reference points (Nz, Cz, AL, RL) and record the coordinates of all optodes. These coordinates were transformed into Montreal Neurological Institute (MNI) space via the NIRS-SPM toolbox. Based on the probabilistic Brodmann area atlas, channels were assigned to specific functional regions, including dorsolateral prefrontal cortex (DLPFC), Broca's area, primary motor cortex (M1), supplementary motor area, primary somatosensory cortex, Wernicke's area, temporal cortex, and visual cortex. The predefined regions of interest (ROIs) for this study included the prefrontal cortex (PFC), DLPFC, sensorimotor cortex (SMC), M1, premotor and supplementary motor areas (pSMA), auditory cortex (AC), and visual cortex (VC). Corresponding Brodmann areas and channels assignments for each ROI are detailed in Table 1 and illustrated in Figure 2.

**Table 1:** Cortical representations of ROIs based on BA and corresponding fNIRS channels.

**ROI**	**BA**	**Channel number**	
		**Ipsilesional hemisphere**	**Contralesional hemisphere**
PFC	8, 10	1, 2, 7, 33, 41, 42	3, 4, 9, 35, 43, 44
DLPFC	9, 45, 46	5, 6, 12, 13, 16, 17, 18, 19, 26, 27, 32, 31	10, 11, 14, 15, 21, 22, 23, 24, 28, 29, 36, 37
SMC	1, 2, 3, 7	60, 61, 83	66, 67, 86
M1	4	50, 84	56, 85
pSMA	6	39, 40, 51, 52, 62, 63, 72, 73	45, 46, 54, 55, 64, 65, 75, 76
AC	21, 22, 38, 40, 43, 48	25, 38, 48, 49, 59, 69, 70, 71, 80, 81, 82	30, 47, 57, 58, 68, 77, 78, 79, 87, 88, 89
VC	17, 18, 19	90, 91, 94, 95, 99, 100, 103, 104	92, 93, 97, 98, 101, 102, 105, 106

ROI, region of interest; BA, Brodmann area; PFC, prefrontal cortex; DLPFC, dorsolateral prefrontal cortex; SMC, sensorimotor cortex; M1, primary motor cortex; pSMA, premotor and supplementary motor areas; AC, auditory cortex; VC, visual cortex.

**Figure 3:**
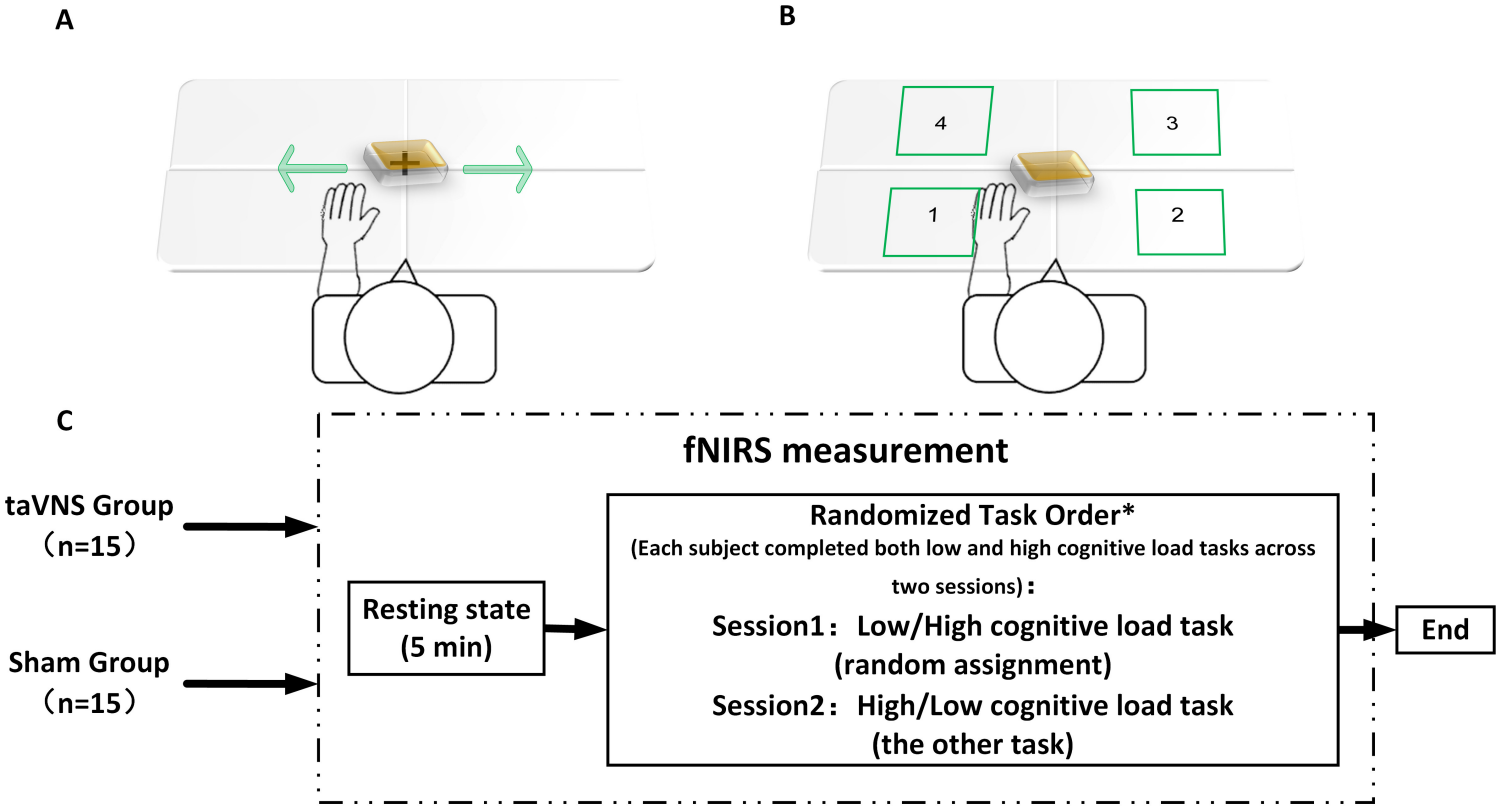
fNIRS evaluation flowchart. **(A)** Low-cognitive-load task; **(B)** high-cognitive-load task; **(C)** fNIRS detection process. *Each subject completed both low and high cognitive load tasks across two sessions, with randomized task assignment ensuring Session 1 and Session 2 always involved opposing cognitive load conditions (i.e., low → high or high → low sequence per subject).

Experiments were conducted in a controlled environment with attenuated ambient illumination and acoustic isolation. Procedures were administered by technicians trained in fNIRS instrument operation, and the entire experimental protocol was guided by computer-generated auditory cues. During the initial phase, a 5-min baseline fNIRS signal was recorded while participants rested with their eyes closed. Subsequently, the computer prompted participants to perform both motor tasks with differential cognitive loads using their hemiparetic hand in two separate sessions (Session 1 and Session 2). The assignment of cognitive load conditions (low/high) to sessions was counterbalanced across participants using a pseudorandomized sequence (Figure 3C), ensuring all participants completed both conditions. Both tasks employed a block design comprising 5 blocks. For the low-cognitive-load task (Figure 3A), each block consisted of a 20-s task execution period followed by a 30-s rest interval. During task execution, participants were instructed to continuously translate a wooden block horizontally from side to side as rapidly and steadily as possible. for the high-cognitive-load task (Figure 3B), each block also comprised a 20-s execution period and a 30-s rest period. Throughout the execution period, the computer sequentially presented randomized auditory number commands (integers 1–4). Following each command, participants were allotted 2 s to move the block and place it into the corresponding numbered target quadrant on the table. Each execution block contained 10 randomized number commands. If a participant failed to complete the movement corresponding to the current command within the 2-s timeframe due to insufficient speed or other unforeseen circumstances, that specific command was discarded, and the next command proceeded immediately. Response accuracy and timeliness for each command were recorded. Completion rate per task block was calculated based on the percentage of commands accurately completed within the allotted time; this metric served as a criterion for determining block inclusion in subsequent neural activation analyses. Continuous fNIRS signal acquisition throughout task execution enabled comparative assessment of cortical activation patterns across cognitive load conditions and evaluation of intervention effects on functional hemodynamics in task-relevant regions.

## 3 Data processing

### 3.1 fNIRS signal processing

Raw fNIRS data were preprocessed utilizing the Homer2 toolbox within MATLAB R2013b (MathWorks, USA), following these sequential procedures (Kamran et al., 2016): (1) conversion of raw light intensity to optical density (OD); (2) detection and correction of motion artifacts; (3) band-pass filtering; (4) conversion of OD to oxyhemoglobin (HbO2) concentration based on the modified Beer-Lambert law; and (5) extraction of the mean HbO2 concentration during the rest period for assessing inter-group baseline homogeneit. To standardize hemispheric alignment and lesion localization, fNIRS channel data from patients with right-hemispheric lesions underwent mirror-flipping, ensuring consistent correspondence of the ipsilesional hemisphere to the left hemisphere across all subjects. The amplitude of low-frequency fluctuation (ALFF) (Hu et al., 2024) was employed to assess the intensity of spontaneous regional neural activity during the resting state. For the task state, regional activation strength was characterized by calculating beta values (β) for each ROI via the general linear model (GLM) implemented within the NIRS_KIT toolbox (Li et al., 2020). To enhance activation signal specificity, response accuracy for each command during the high-cognitive-load motor task was recorded; only blocks achieving a completion rate ≥80% within the task period were incorporated into the GLM analysis, thereby controlling for confounding effects of insufficient task execution. Furthermore, considering the significant time-varying characteristics of neural activity during tasks, functional connectivity analysis in the task state utilized wavelet coherence (Hakim et al., 2023), which offers superior time-frequency sensitivity, to construct functional connectivity matrices between ROIs. In contrast, due to the relative stability of neural activity during rest, the resting-state functional connectivity matrix was derived using the phase locking value (PLV) (Li et al., 2021b), a measure sensitive to phase synchronization and well-suited for low-frequency signals, reflecting the relative synchrony between ROIs. To further investigate the topological properties of neural functional networks, the resultant resting-state and task-state functional connectivity matrices were imported into the Gretna toolbox for complex network analysis. Computed network metrics included nodal clustering coefficient, nodal local efficiency, and global efficiency. To circumvent potential bias associated with single-threshold selection in network structure analysis, a sparsity-based thresholding method (Wu et al., 2024) was applied to regulate network connection density, with the threshold spanning 0.10–0.50 in increments of 0.05. Finally, the area under the curve (AUC) for each network metric across this threshold range was calculated to facilitate robust comparison of topological properties across different experimental conditions.

### 3.2 Statistical analysis

All statistical analyses were conducted in Jamovi (version 2.4.8) (JAMOVI, 2023). Normality of continuous variables was assessed using the Shapiro-Wilk test. For baseline demographic and clinical characteristics, group comparisons of categorical variables were conducted using Fisher's exact test, while continuous variables were compared using independent samples *t*-tests for normally distributed data or Mann-Whitney U tests for non-normally distributed data. Between-group comparisons of behavioral outcomes (FMA-UE, MoCA, FSS, MBI), HRV parameters (HR, SDNN, RMSSD, LF/HF ratio), and MEP parameters (amplitude, latency) were conducted using analysis of covariance (ANCOVA) with group assignment as the between-subject factor, post-intervention change scores as the dependent variable, and corresponding baseline values as covariates. Within-group comparisons employed paired samples *t*-tests or Wilcoxon signed-rank tests based on data normality. Changes in the ipsilesional MEP elicitation rate were analyzed using the McNemar test for within-group comparisons and Fisher's exact test for between-group comparisons. For fNIRS data, between-group comparisons of resting-state spontaneous neural activity (ALFF), task-related regional activation intensity (β), and complex network topology metrics (nodal clustering coefficient, nodal local efficiency, global efficiency) were performed via ANCOVA, using post-intervention values as the dependent variable and baseline values as covariates. Results are presented as least squares means (LS Means) with corresponding 95% confidence intervals (95% CI), derived from estimated marginal means (EMM). Between-group comparisons of resting-state and task-state functional connectivity metrics were conducted using independent samples *t*-test or Mann-Whitney *U* tests according to data normality. Within-group changes were assessed by paired samples *t*-test or Wilcoxon signed-rank tests as appropriate. The False Discovery Rate (FDR) correction was applied for multiple comparisons, with statistical significance set at *P* < 0.05. Finally, between-group comparison results for resting-state spontaneous activity, task-related regional activation, and functional connectivity were visualized in 3D using the BrainNet Viewer toolbox.

## 4 Results

### 4.1 Demographic and clinical characteristics

The demographic and baseline clinical characteristics of participants in both groups are presented in Table 2. No statistically significant intergroup differences were observed in age, sex distribution, stroke duration, stroke etiology, side of hemiparesis, FMA-UE scores, or MoCA scores at baseline (*P* > 0.05).

**Table 2:** Baseline demographic and clinical characteristics of patients between the two groups.

**Variables**	**taVNS group (*n* = 15)**	**Sham group (*n* = 15)**	***t***	***P***
**Demographics**				
Age (years, mean ± SD)	50.33 ± 14.41	51.07 ± 13.68	−0.143	0.887^a^
Gender (male/female, *n*)	8/7	12/3	–	0.128^b^
**Stroke characteristics**				
Stroke type (hemorrhagic/ischemic)	5/10	9/6	–	0.272^b^
Stroke onset (days, mean ± SD)	124.47 ± 23.03	106.67 ± 33.07	1.711	0.098^a^
Hemiparetic side (left/right, *n*)	8/7	6/9	–	0.715^b^
**Baseline functional scores**				
FMA-UE (score, mean ± SD)	38.53 ± 4.37	42.93 ± 9.38	−1.646	0.111^a^
MoCA (score, mean ± SD)	20.67 ± 1.99	22.13 ± 2.72	−1.685	0.103^a^

SD, standard deviation; FMA-UE, Fugl-Meyer assessment-upper extremity; MoCA, montreal cognitive assessment.

*a*Independent samples t-test;

*b*Fisher's exact test.

### 4.2 HRV outcomes

Significant post-intervention improvements in HR, SDNN, RMSSD, and LF/HF ratio were demonstrated in the taVNS group compared to baseline (*P* < 0.05). In contrast, the Sham group exhibited only significant HR reduction (*P* < 0.05). The improvement in all HRV indices was significantly greater in the taVNS group vs. the Sham group (*P* < 0.05), with detailed data presented in Table 3.

**Table 3:** Comparison of HRV post-intervention between the two groups.

**Variables**	**Group**	**Descriptive analysis**		**Between-group differences (VNS-Sham, ANCOVA)**			
		**T0** **Mean (SD)**	**T1 Mean (SD)**	**Differences in LS mean (95% CI)**	***P***	***F***	η^2^
HR	taVNS	85.67 (12.93)	78.00 (11.61)^***^	−4.321 (−6.951, −1.692)	0.002	11.410	0.305
	Sham	86.53 (15.49)	83.07 (13.67)^*^				
SDNN	taVNS	18.40 (4.38)	25.95 (7.21)^***^	5.594 (0.960,10.228)	0.020	6.157	0.191
	Sham	14.98 (4.39)	17.27 (6.33)				
RMSSD	taVNS	16.12 (4.89)	21.18 (7.69)^***^	4.736 (0.957,8.514)	0.016	6.637	0.203
	Sham	12.66 (5.83)	13.41 (5.42)				
LF/HF	taVNS	1.21 (0.44)	0.75 (0.33)^**^	−0.359 (−0.628, −0.090)	0.011	7.548	0.225
	Sham	1.19 (0.41)	1.10 (0.44)				

ANCOVA, analysis of covariance; T0, baseline; T1, after 3 weeks of treatment; SD, standard deviation; HRV, heart rate variability; LS, least squares; CI, confidence interval; HR, average heart rate; SDNN, standard deviation of the normal-to-normal (NN) intervals; RMSSD, square root of the mean squared differences of successive NN intervals; LF/HF, the ratio of low-frequency to high-frequency power;

* p < 0.05,

** p < 0.01,

*** p < 0.001 vs. T0.

### 4.3 FMA-UE, MoCA, MBI, and FSS outcomes

Both groups showed significant within-group improvements in FMA-UE, MoCA, MBI, and FSS scores post-intervention compared to baseline (*P* < 0.05). However, the taVNS group demonstrated significantly superior improvements across all behavioral metrics compared to the Sham group (*P* < 0.05). Comprehensive results are provided in Table 4.

**Table 4:** Comparison of post-intervention clinical scale scores between the two groups.

**Variables**	**Group**	**Descriptive analysis**		**Between-group differences (VNS-Sham, ANCOVA)**			
		**T0** **Mean (SD)**	**T1 Mean (SD)**	**Differences in LS mean (95% CI)**	***P***	***F***	η^2^
FMA-UE	taVNS	38.53 (4.37)	50.27 (6.90)^***^	8.453 (4.472,12.163)	0.000	21.923	0.457
	Sham	42.93 (9.38)	46.67 (10.97)^**^				
MoCA	taVNS	20.67 (1.99)	25.87 (2.42)^**^	3.236 (1.593,4.880)	0.000	16.382	0.387
	Sham	22.13 (2.72)	23.33 (2.53)^*^				
MBI	taVNS	46.33 (28.12)	54.67 (29.54)^***^	5.636 (2.177,9.096)	0.002	11.213	0.301
	Sham	69.33 (27.51)	71.67 (25.82)^*^				
FSS	taVNS	45.67 (7.74)	38.60 (8.50)^***^	−2.926 (−5.501, −0.351)	0.027	5.456	0.173
	Sham	46.73 (7.51)	42.47 (6.45)^***^				

ANCOVA, analysis of covariance; T0, baseline; T1, after 3 weeks of treatment; SD, standard deviation; LS, least squares; CI, confidence interval; FMA-UE, Fugl-Meyer assessment-upper extremity; MoCA, montreal cognitive assessment; MBI, modified barthel index; FSS, fatigue severity scale;

* p < 0.05,

** p < 0.01,

*** p < 0.001 vs. T0.

### 4.4 MEPs outcomes

The taVNS group exhibited a significant increase in ipsilesional MEP elicitation rate post-intervention vs. baseline (*P* < 0.05), with this increase being significantly greater than observed in the Sham group (*P* < 0.05). Additionally, the taVNS group showed significant reduction in contralesional MEP latency and amplitude enhancement (*P* < 0.05). The Sham group demonstrated only significant contralesional amplitude improvement (*P* < 0.05), with no significant change in latency. The reduction in contralesional MEP latency was significantly greater in the taVNS group than the Sham group (*P* < 0.05). Detailed results are presented in Table 5.

**Table 5:** **(A)** Comparison of contralesional MEP amplitude and latency post-intervention between the two groups. **(B)** Comparison of ipsilesional MEP elicitation rates post-intervention between the two groups.

**(A)**							
**Variables**	**Group**	**Descriptive analysis**		**Between-group differences (VNS-Sham, ANCOVA)**			
		**T0 (elicited/not elicited)**	**T1 (elicited/not elicited)**	***P***			
Ipsilesional MEP elicitation rate	taVNS	3/12	10/5^*^	0.025			
	Sham	1/14	3/12				
**(B)**							
**Variables**	**Group**	**Descriptive analysis**		**Between-group differences (VNS-Sham, ANCOVA)**			
		**T0** **Mean (SD)**	**T1** **Mean (SD)**	**Differences in LS mean (95% CI)**	***P***	***F***	η^2^
Contralesional MEP amplitude	taVNS	63.90 (24.65)	124.07 (40.54)^***^	22.854 (−8.782,54.490)	0.150	2.205	0.078
	Sham	110.93 (66.09)	127.93 (63.97)^*^				
Contralesional MEP latency	taVNS	41.37 (10.03)	24.67 (4.99)^***^	−9.304 (−14.226, −4.382)	0.001	15.097	0.367
	Sham	30.85 (6.08)	32.33 (5.50)				

MEP, motor-evoked potential; ANCOVA, analysis of covariance; T0, baseline; T1, after 3 weeks of treatment; SD, standard deviation; LS, least squares; CI, confidence interval;

* p < 0.05,

** p < 0.01,

*** p < 0.001 vs. T0.

### 4.5 fNIRS outcomes

#### 4.5.1 Resting-state spontaneous neural activity (ALFF)

**Figure 4:**
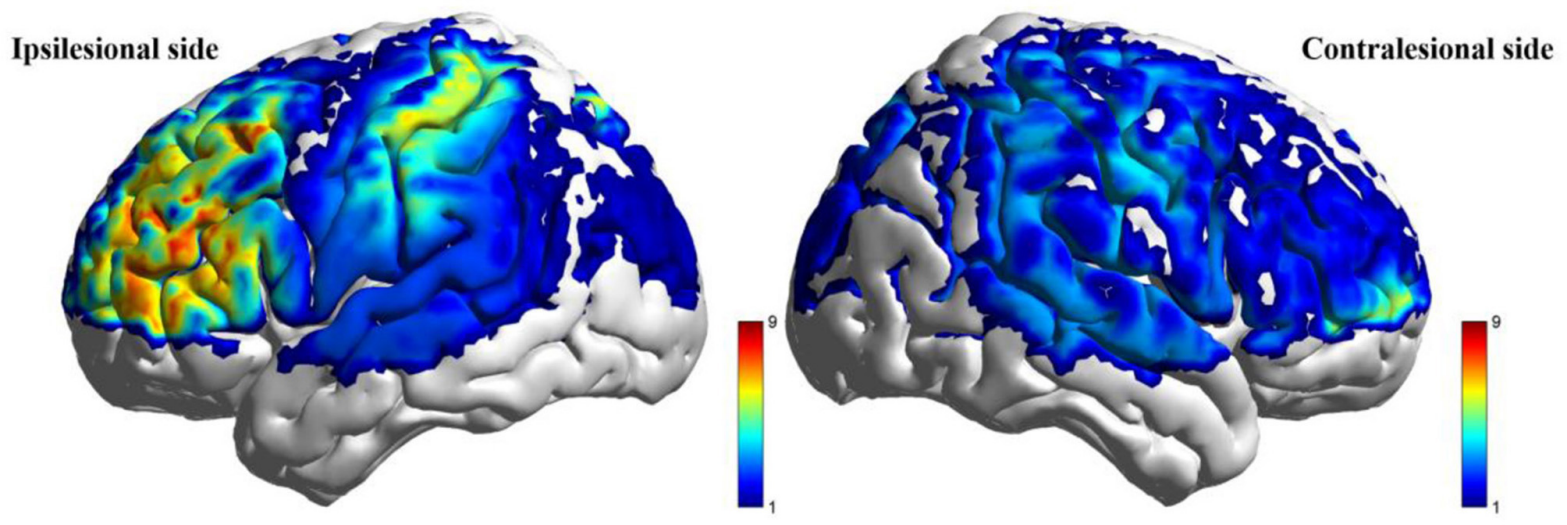
Between-group comparison of ALFF during resting state after intervention (taVNS-Sham).

Post-intervention resting-state analysis revealed significantly higher ALFF values in the taVNS group vs. the Sham group within the ipsilesional PFC, DLPFC, and SMC (*P* < 0.05). However, these regional differences did not retain statistical significance after FDR correction (*P*_FDR_ > 0.05). No statistically significant within-group changes in ALFF were observed from baseline to post-intervention in either cohort (*P* > 0.05). Detailed results are presented in Table 6 and Figure 4.

**Table 6:** Comparison of resting-state ALFF post-intervention between the two groups.

**ROI**	**Group**	**Descriptive analysis**		**Between-group differences (VNS-Sham, ANCOVA)**		
		**T0** **Mean (SD)**, × **10**^−7^	**T1** **Mean (SD)**, × **10**^−7^	***F***	***P***	*P*_FDR_
iPFC	taVNS	−1.698 (2.725)	1.274 (2.114)	6.429	0.017	0.0793
	Sham	−0.391 (1.677)	−0.929 (1.207)			
iDLPFC	taVNS	−0.154 (3.438)	1.615 (1.934)	8.164	0.008	0.0793
	Sham	−0.872 (2.463)	−0.852 (1.070)			
iSMC	taVNS	−2.566 (3.928)	0.334 (4.415)	6.722	0.015	0.0793
	Sham	−2.969 (5.543)	−1.063 (1.858)			

ALFF, amplitude of low-frequency fluctuation; ANCOVA, analysis of covariance; T0, baseline; T1, after 3 weeks of treatment; SD, standard deviation; iPFC, ipsilesional prefrontal cortex; iDLPFC, ipsilesional dorsolateral prefrontal cortex; iSMC, ipsilesional sensorimotor cortex; FDR, false discovery rate.

#### 4.5.2 Task-related brain activation changes

**Figure 5:**
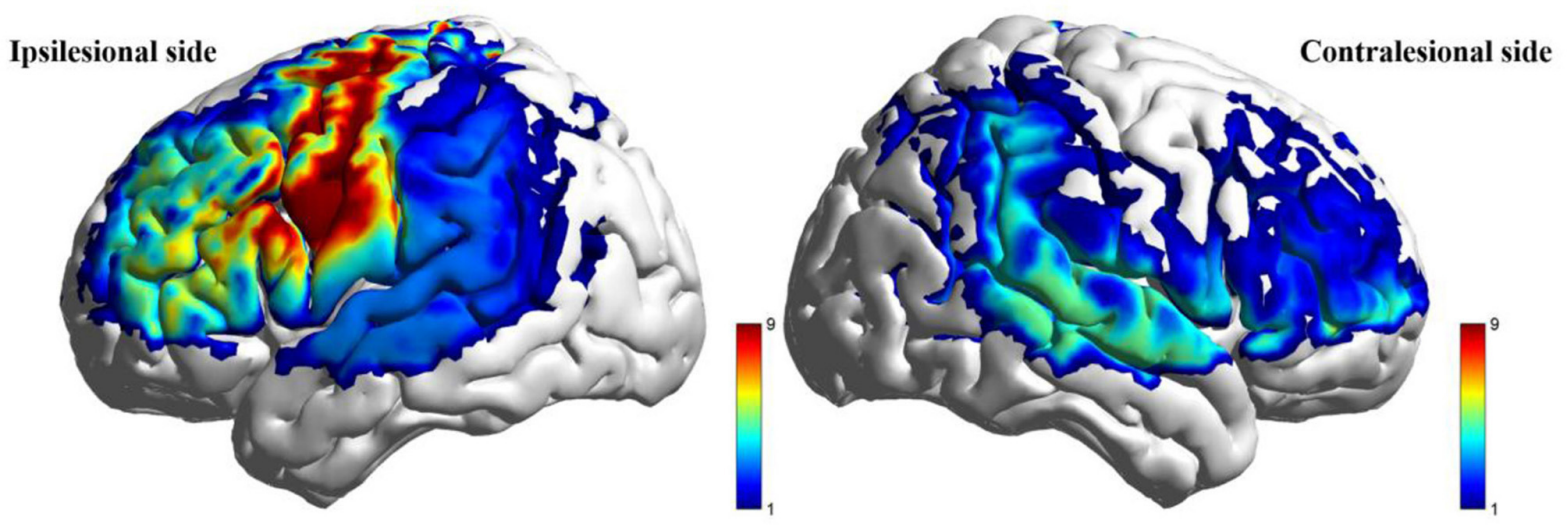
Between-group comparison of beta value during low-cognitive-load motor task after intervention (taVNS-Sham).

**Figure 6:**
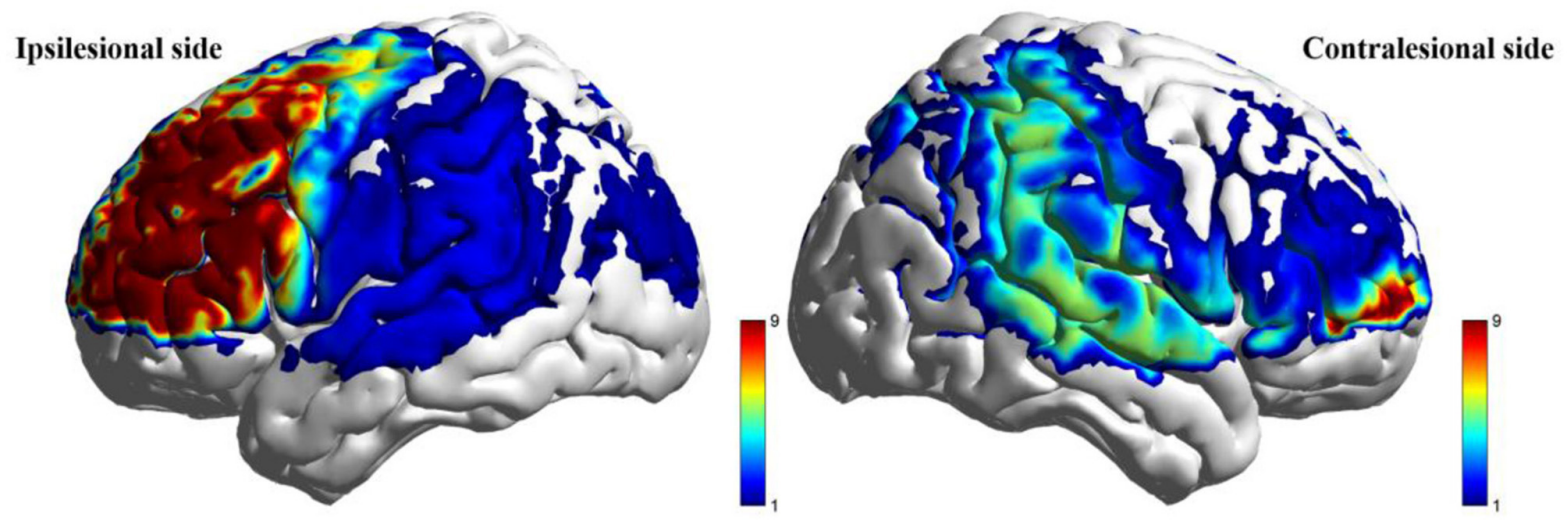
Between-group comparison of beta value during high-cognitive-load motor task after intervention (taVNS-Sham).

During the low-cognitive-load motor task, general linear model (GLM) analysis demonstrated significantly higher beta values, reflecting activation strength, in the taVNS group compared to the Sham group within the ipsilesional DLPFC, M1, and pSMA, as well as the contralesional AC (*P* < 0.05). Following FDR correction, activation differences in the ipsilesional M1 and pSMA remained significant (*P*_FDR_ > 0.05; Table 7, Figure 5). For the high-cognitive-load motor task, the taVNS group demonstrated significantly elevated activation levels than the Sham group in the ipsilesional PFC, DLPFC, and pSMA, alongside the contralesional VC and SMC (*P* < 0.05). Following FDR correction, beta value differences in the ipsilesional PFC and DLPFC retained significance (*P*_FDR_ > 0.05). Within-group analyses identified that only the taVNS group showed significant post-intervention increases in ipsilesional PFC activation during the high-cognitive-load task compared to baseline (*P*_FDR_ > 0.05). No other regions exhibited significant longitudinal changes in either group (Table 8, Figure 6).

**Table 7:** Comparison of beta values during low-cognitive-load motor tasks post-intervention between the two groups.

**ROI**	**Group**	**Descriptive analysis**		**Between-group differences (VNS-Sham, ANCOVA)**		
		**T0** **Mean (SD)**, × **10**^−7^	**T1 Mean (SD)**, × **10**^−7^	***F***	***P***	*P*_FDR_
iDLPFC	taVNS	−3.135 (6.213)	1.913 (5.031)	6.542	0.017	0.079
	Sham	5.753 (3.529)	−2.435 (5.088)			
iM1	taVNS	−2.606 (8.891)	6.384 (5.518)	15.401	0.001	0.014
	Sham	1.016 (9.412)	−6.485 (1.109)			
ipSMA	taVNS	−2.614 (6.311)	1.509 (4.862)	12.268	0.002	0.014
	Sham	−2.765 (8.968)	−5.132 (5.523)			
cAC	taVNS	−2.889 (8.676)	7.725 (2.288)	4.801	0.038	0.133
	Sham	1.443 (9.133)	−5.805 (1.091)			

ANCOVA, analysis of covariance; T0, baseline; T1, after 3 weeks of treatment; SD, standard deviation; iDLPFC, ipsilesional dorsolateral prefrontal cortex; iM1, ipsilesional primary motor cortex; ipSMA, ipsilesional premotor and supplementary motor areas; cAC, contralesional auditory cortex; FDR, false discovery rate.

**Table 8:** Comparison of beta values during high-cognitive-load motor tasks post-intervention between the two groups.

**ROI**	**Group**	**Descriptive analysis**		**Between-group differences (VNS-Sham, ANCOVA)**		
		**T0** **Mean (SD)**, × **10**^−7^	**T1** **Mean (SD)**, × **10**^−7^	***F***	***P***	*P*_FDR_
iPFC	taVNS	−1.698 (2.725)	1.274 (2.114)^**^	14.483	0.001	0.007
	Sham	−0.391 (1.677)	−0.929 (1.207)			
iDLPFC	taVNS	−0.154 (3.438)	1.615 (1.934)	17.723	0.000	0.000
	Sham	−0.872 (2.463)	−0.852 (1.070)			
ipSMA	taVNS	1.017 (2.586)	1.047 (3.556)	7.420	0.011	0.053
	Sham	7.031 (3.334)	1.806 (1.613)			
cSMC	taVNS	−1.975 (10.810)	5.771 (3.075)	5.228	0.031	0.087
	Sham	3.127 (14.160)	−1.675 (2.223)			
cAC	taVNS	−1.504 (8.066)	0.989 (3.640)	5.195	0.031	0.087
	Sham	−2.170 (5.409)	−3.064 (5.495)			

ANCOVA, analysis of covariance; T0, baseline; T1, after 3 weeks of treatment; SD, standard deviation; iPFC, ipsilesional prefrontal cortex; iDLPFC, ipsilesional dorsolateral prefrontal cortex; ipSMA, ipsilesional premotor and supplementary motor areas; cSMC, contralesional sensorimotor cortex; cAC, contralesional auditory cortex; FDR, false discovery rate;

** p < 0.01 vs. T0.

#### 4.5.3 Resting-state and task-state network connectivity

**Figure 7:**
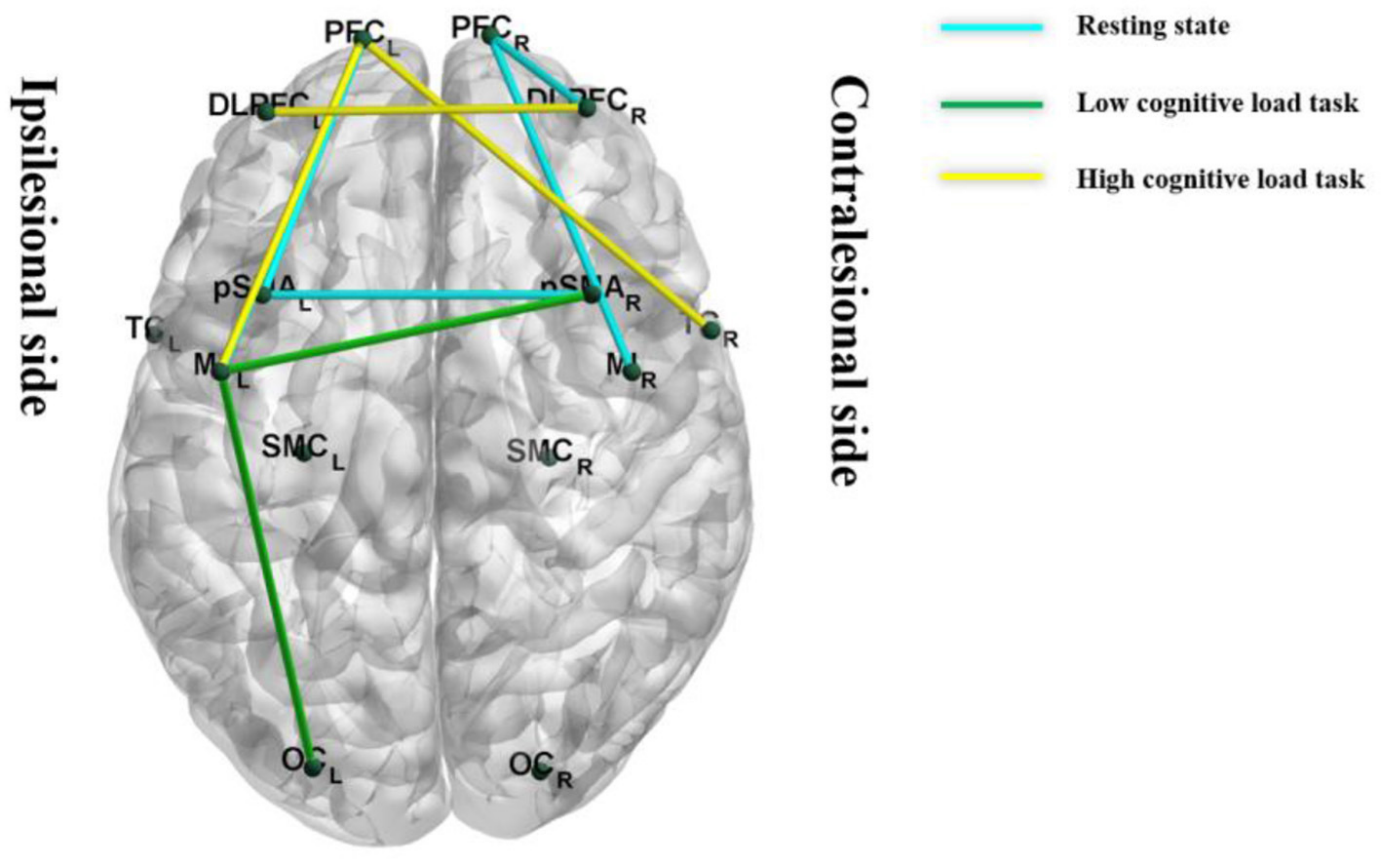
Between-group comparison of functional connectivity under different conditions (taVNS-Sham). All displayed connections survived the uncorrected threshold of *P* < 0.05 (independent samples *t*-test). No connections retained statistical significance following false discovery rate (FDR) correction.

Both resting-state and task-state data showed nominally increased intra-/inter-hemispheric functional connectivity strength in the taVNS group (*P* < 0.05, Figure 7), though these differences were non-significant post-FDR correction. Complex network analysis further indicated that during resting state, the taVNS group showed a trend of increased nodal clustering coefficient in the ipsilesional DLPFC compared to the Sham group (*P* < 0.05). During the low-cognitive-load motor task, the taVNS group demonstrated increased nodal clustering coefficients in the ipsilesional M1 and contralesional pSMA and increased nodal local efficiency in the ipsilesional pSMA compared to the Sham group (*P* < 0.05). During the high-cognitive-load motor task, increased nodal clustering coefficient was observed in the ipsilesional DLPFC and VC, and contralesional AC, along with increased nodal local efficiency in the ipsilesional PFC and DLPFC, and increased global efficiency in the contralesional DLPFC and VC in the taVNS group compared to Sham (*P* < 0.05). After FDR correction, only the differences in nodal clustering coefficient and nodal local efficiency of the ipsilesional DLPFC during the high-cognitive-load task remained statistical significance (*P*_FDR_ < 0.05). Detailed results are presented in Table 9.

**Table 9:** Comparison of network parameters post-intervention between the two groups.

**Network parameters**	**ROI**	**Group**	**Descriptive analysis**		**Between-group differences (VNS-Sham, ANCOVA)**		
			**T0** **Mean (SD)**	**T1** **Mean (SD)**	***F***	***P***	*P*_FDR_
**Resting state**							
Nodal clustering coefficient	iDLPFC	taVNS	0.177 (0.136)	0.181 (0.129)	5.102	0.032	0.448
		Sham	0.220 (0.083)	0.247 (0.098)			
Nodal local efficiency	No significant difference						
Global efficiency	No significant difference						
**Low cognitive load motor task**							
Nodal clustering coefficient	iM1	taVNS	0.272 (0.091)	0.349 (0.085)^*^	7.538	0.011	0.154
		Sham	0.261 (0.080)	0.251 (0.105)			
	ipSMA	taVNS	0.292 (0.106)	0.307 (0.093)	4.600	0.041	0.287
		Sham	0.259 (0.135)	0.214 (0.135)			
Nodal local efficiency	ipSMA	taVNS	0.255 (0.127)	0.343 (0.089)	5.577	0.026	0.364
		Sham	0.249 (0.136)	0.265 (0.114)			
Global efficiency	No significant difference						
**High cognitive load motor task**							
Nodal clustering coefficient	iDLPFC	taVNS	0.208 (0.096)	0.371 (0.037)^***^	16.235	0.000	0.000
		Sham	0.335 (0.020)	0.309 (0.031)^**^			
	iVC	taVNS	0.245 (0.133)	0.178 (0.142)	5.058	0.033	0.154
		Sham	0.280 (0.117)	0.280 (0.090)			
	cAC	taVNS	0.290 (0.063)	0.231 (0.135)	5.071	0.033	0.154
		Sham	0.313 (0.079)	0.322 (0.077)			
Nodal local efficiency	iPFC	taVNS	0.256 (0.128)	0.372 (0.020)^**^	8.340	0.008	0.056
		Sham	0.312 (0.070)	0.315 (0.079)			
	iDLPFC	taVNS	0.251 (0.119)	0.376 (0.028)^**^	13.251	0.001	0.014
		Sham	0.360 (0.016)	0.312 (0.043)^***^			
Global efficiency	cDLPFC	taVNS	0.207 (0.048)	0.205 (0.047)	5.237	0.030	0.259
		Sham	0.215 (0.041)	0.148 (0.083)			
	cVC	taVNS	0.192 (0.057)	0.155 (0.066)	4.831	0.037	0.259
		Sham	0.160 (0.076)	0.208 (0.050)			

ANCOVA, Analysis of Covariance; T0, baseline; T1, after 3 weeks of treatment; SD, standard deviation; iPFC, ipsilesional prefrontal cortex; i/cDLPFC, ipsilesional/contralesional dorsolateral prefrontal cortex; iM1, ipsilesional primary motor cortex; ipSMA, ipsilesional premotor and supplementary motor areas; cAC, contralesional auditory cortex; cVC, contralesional visual cortex; FDR, false discovery rate;

* p < 0.05,

** p < 0.01,

*** p < 0.001 vs. T0.

## 5 Discussion

Recent research has increasingly emphasized non-invasive brainstem neuromodulation techniques, particularly taVNS, as a promising intervention for post-stroke functional rehabilitation. Compared to invasive vagus nerve stimulation, taVNS offers distinct advantages including non-surgical administration, enhanced patient compliance, and the capacity for flexible integration with rehabilitation tasks during execution, thereby enabling a real-time “stimulation-task synergy” mechanism. Its superior safety profile, scalability, and cost-effectiveness further support clinical translation (Badran et al., 2023; Shi et al., 2023). Within the broader landscape of non-invasive brain stimulation (NIBS), techniques such as transcranial magnetic stimulation (TMS) and transcranial direct current stimulation (tDCS) have also been investigated for stroke rehabilitation, but their clinical adoption remains limited by variable protocols, heterogeneity of patient response, and logistical demands (Yokota et al., 2022; Balderston et al., 2022). In contrast, taVNS engages neuromodulatory systems indirectly via vagal afferent pathways, allowing for peripheral administration and concurrent integration with functional tasks (Lee et al., 2025). This distinct mechanism may complement the corticospinal and cortical modulation achieved by TMS and tDCS, potentially offering synergistic effects in future combined approaches. Relative to conventional rehabilitation, taVNS demonstrates not only favorable safety and reproducibility but also unique potential for modulating multisystem neural circuits, enhancing cortical plasticity, autonomic homeostasis, and executive functions (Jonker et al., 2021; Balderston et al., 2022; Lee et al., 2025; Camargo et al., 2024; Forte et al., 2022; Kang et al., 2024). Although existing studies indicate taVNS-mediated improvements in motor function at the behavioral level, its integrated neuromodulatory mechanisms, particularly the cortical dynamic response patterns under cognitive modulation, remain systematically unverified (Wang et al., 2024b; Gianlorenco et al., 2022). This study therefore aimed to investigate the neurofunctional benefits of taVNS-paired TOT in stroke patients with hemiplegia using multimodal metrics, focusing specifically on cortical response patterns to taVNS modulation under varying cognitive loads.

Employing HRV as a physiological index of sympathovagal balance (Kang et al., 2024), we observed significantly increased SDNN and RMSSD with concomitant reductions in HR and LF/HF ratio in the taVNS group post-intervention. These findings indicate enhanced parasympathetic activity and reduced sympathetic tone, confirming taVNS successfully activated vagal pathways and improved autonomic nervous system regulation, consistent with prior research (Owens et al., 2024), which demonstrate that taVNS activates medullary vagal pathways, inducing systemic parasympathetic excitation to improve cardiovascular autonomic control. Crucially, established research (Zou et al., 2024) indicates that establishing autonomic homeostasis provides essential support for neural plasticity and motor learning processes, further substantiating our observations.

MEPs were assessed in both groups pre- and post-intervention to evaluate taVNS effects on corticospinal tract plasticity. Results demonstrated a significant increase in ipsilesional MEP elicitation rate within the taVNS group compared to baseline, with the improvement magnitude significantly exceeding that of the Sham group. Additionally, the taVNS group exhibited significantly shortened MEP latency and increased amplitude in the contralesional hemisphere. These results suggest taVNS effectively activates impaired neural pathways and enhances excitability in contralesional pathways, reflecting its synergistic modulation of bilateral corticospinal motor tracts. This mechanism may relate to taVNS promoting cortical synaptic activity and increasing neuronal excitability, thereby enhancing cortical output efficiency to the spinal cord. Notably, improved ipsilesional MEP elicitation rates may indicate enhanced neural recruitment capacity within the corticospinal pathways, while contralesional latency and amplitude changes suggest improved functional efficiency of existing conduction pathways (Yun et al., 2025; van Midden et al., 2023). Collectively, taVNS promotes reorganization and functional recovery of damaged neural networks by boosting excitability and elicitation rates in the damaged cortex (Wang et al., 2024b; Badran et al., 2023). Concurrently, it induces compensatory excitation in the contralesional hemisphere, not only strengthening its inherent compensatory functions but also potentially supporting recovery in the ipsilesional cortex via transhemispheric regulatory mechanisms (Li et al., 2025; Huang et al., 2023). Crucially, the pattern of changes—increased excitability in the ipsilesional hemisphere alongside reduced latency in the contralesional hemisphere—may reflect a modulation of interhemispheric inhibitory dynamics. This could indicate a reduction in excessive inhibition from the contralesional hemisphere onto the ipsilesional hemisphere, a key mechanism of interhemispheric imbalance implicated in post-stroke motor impairment (Garrido et al., 2023). While direct measures of interhemispheric inhibition were not obtained, this MEP profile provides indirect support for the hypothesis that taVNS contributes to restoring a more balanced interhemispheric interaction, alongside enhancing excitability within the lesioned pathways. This dual mechanism—enhancing reconstruction capacity in impaired pathways while optimizing compensatory efficacy in contralesional pathways—holds promise for synergistically remodeling higher-order motor control networks, offering a potentially more effective intervention strategy for central nervous system functional recovery.

fNIRS results further elucidated taVNS mechanisms within cognitive-motor integration. While resting-state analyses showed a trend toward higher spontaneous neural activity in the ipsilesional PFC, DLPFC, and SMC cortices in the taVNS group, these differences were non-significant post-FDR correction. This suggests unstable intergroup effects on regional activity at rest, potentially limited by substantial individual variability and signal fluctuation (Yokota et al., 2022; Keatch et al., 2025). In contrast, taVNS-induced activation patterns during tasks were more focused and stable, exhibiting distinct network responses across cognitive loads. During the low-cognitive-load motor task, the taVNS group demonstrated elevated activation in the ipsilesional DLPFC, M1, pSMA, and the contralesional AC. Activation increases in M1 and pSMA remained statistically significant after multiple comparisons correction. The sustained significant activation of these core motor hubs (M1, pSMA) suggests taVNS may accelerate action generation and execution by enhancing initiation and synergistic control mechanisms (Wang et al., 2024b; Gerges et al., 2025). Co-activation of DLPFC and AC also indicates taVNS potentially facilitates attentional modulation and movement preparation processes, potentially enhancing motor cortical responses indirectly by improving premotor cognitive engagement (Li et al., 2023; Harrison et al., 2025). During the high-cognitive-load motor task, regions showing enhanced activation in the taVNS group expanded to include the ipsilesional PFC, DLPFC, pSMA, and contralesional VC and SMC, with PFC and DLPFC exhibiting the most significant increases. As key regions for higher-order cognitive control and motivational drive, their sustained significant activation under complex task demands suggests taVNS may enhance executive efficiency and goal-directedness by boosting the involvement of advanced cognitive control and motivational systems (An et al., 2025). This interpretation is reinforced by within-group analyses demonstrating significant post-intervention increases specifically in ipsilesional PFC activation among taVNS participants, identifying this region as a critical node for taVNS modulation during complex cognitive-motor tasks. Furthermore, the concurrent involvement of pSMA, SMC, and VC implies taVNS plays a significant role in strengthening overall motor regulation and mediating “cognition-driven motor cortex activation”. Importantly, the activation pattern observed in the contralesional hemisphere during both task loads—characterized by co-activation (AC in low-load) or supplementary activation (SMC, VC in high-load)—aligns with the notion that taVNS may promote a more balanced and cooperative interhemispheric engagement (Zhou et al., 2021). This contrasts with patterns of maladaptive contralesional over-activation sometimes observed in stroke (Peng et al., 2023), suggesting taVNS could help attenuate such hemispheric imbalance and foster more efficient bihemispheric collaboration, particularly under cognitively demanding conditions where top-down control is crucial. This activation pattern aligns with the “motor-cognitive fusion model” (Bestmann and Krakauer, 2015) and resembles the prefrontal-motor network synergy enhancement observed in respiratory-gated taVNS studies (Han et al., 2025). Supporting evidence (An et al., 2025) further confirms that taVNS can strengthen DLPFC-PFC functional coupling during high-cognitive-load tasks, thereby improving complex motor task performance efficiency. Beyond motor execution, this enhanced prefrontal connectivity may also underlie the cognitive improvements (e.g., MoCA score increases) and reduced fatigue (FSS score decreases) observed in the taVNS group. Ascending projections from the nucleus tractus solitarius to the locus coeruleus–norepinephrine and basal forebrain cholinergic systems could facilitate attentional regulation, executive control, and arousal stability, thereby improving overall cognitive-motor integration and alleviating fatigue-related performance decline (Giraudier et al., 2022). Although sensory outcomes were not directly assessed in this study, previous evidence suggests that vagal pathway activation may influence thalamocortical sensory processing, highlighting the potential for taVNS to support sensory recovery as part of an integrated rehabilitation strategy. In summary, taVNS elicited distinct regional activation patterns depending on cognitive load: activation dominated by motor hubs with cognitive region co-activation during low-cognitive-load tasks, shifting toward cognitive hub dominance driving broader motor network participation during high-cognitive-load tasks. This suggests that taVNS enhances cognitive-motor integration efficiency by modulating the driving intensity of cognition on motor execution according to task demands. This load-dependent activation profile likely originates from taVNS modulation of the brainstem-prefrontal-motor cortex pathway. By activating the locus coeruleus-norepinephrine system (Horinouchi et al., 2024; Szeska et al., 2025) and prefrontal regions (e.g., DLPFC), taVNS enhances cognitive control capacity and strengthens prefrontal-motor cortical coupling, facilitating a dynamic shift from “motor-dominant” to “cognition-driven” processing based on task complexity (Giraudier et al., 2022; Viglione et al., 2023). The potential modulation of interhemispheric interactions, as suggested by both MEP and fNIRS findings, further underscores taVNS's synergistic regulatory capacity in stroke rehabilitation, potentially promoting higher-order integrative functional recovery through cognitive reinforcement of motor pathways and the restoration of more balanced hemispheric dynamics.

Complex network analysis revealed taVNS-induced dynamic modulation of cortical functional connectivity. Resting-state data showed increased nodal clustering coefficient in the ipsilesional DLPFC in the taVNS group, indicating enhanced local information integration capacity within this region (Luo et al., 2022). Task-state network reorganization exhibited cognitive-load dependency: During the low-cognitive-load task, increased nodal clustering coefficient in ipsilesional M1 and contralesional pSMA, alongside increased nodal local efficiency in ipsilesional pSMA, suggested taVNS enhanced local integration and functional synergy within motor-related regions (Owens et al., 2024). Conversely, during the high-cognitive-load task, network optimization manifested as broad cross-regional reorganization. Specifically, increased nodal clustering coefficient was observed in ipsilesional DLPFC, VC, and contralesional AC; enhanced nodal local efficiency occurred in ipsilesional PFC and DLPFC; and increased global efficiency was found in contralesional DLPFC and VC. These changes indicate that under high-cognitive load, taVNS may enhance overall coordination and resource integration within the prefrontal-motor network by boosting local and inter-regional information transfer efficiency. The consistent DLPFC involvement across multiple metrics positions it as a key hub for taVNS modulation of cognitive-motor integration, aligning with Pereira's “executive control-motor planning” synergy model (Pereira et al., 2024) emphasizing DLPFC's dual role in cognitive control and motor planning during high-demand tasks. Enhanced network efficiency here may reflect superior behavioral regulation capacity. This network reorganization pattern closely matches the “motor activation-dependent neuroplasticity” observed in closed-loop taVNS systems (Zhuang et al., 2023), indicating DLPFC's role as a core network hub participating in the synergistic integration of task control and motor planning (Sommer et al., 2023). Previous studies (Holub et al., 2023; Han et al., 2023; Wheelock et al., 2023; Gondo et al., 2023; Sintini et al., 2024) also found taVNS-induced functional connectivity changes associated with default mode network remodeling, potentially modulating global network states to create an internal environment conducive to neural plasticity. These topological parameter changes suggest taVNS operates via distinct mechanisms across cognitive loads: prioritizing activation and integration of local motor hub networks to enhance execution efficiency during low-cognitive-load tasks, while primarily enhancing connection integration within cognitive hubs (e.g., DLPFC) to guide broad motor region collaboration for optimized resource allocation and system integration during high-cognitive-load tasks. This demonstrates taVNS's flexible adaptation to the cognitive control-motor execution pathway, exhibiting task-load dependency. Clinically, stroke patients frequently exhibit insufficient cognitive resource mobilization and low task control efficiency during functional recovery even without overt cognitive impairment (Potts et al., 2024; Rajda et al., 2025; Bachar Kirshenboim et al., 2025). Our findings suggest that taVNS not only improves motor execution but also possesses the potential to support task regulation and resource integration under increased cognitive load. Consequently, compared to traditional interventions primarily targeting motor cortex activation, taVNS demonstrates the capacity to modulate cognition-driven pathways and promote prefrontal-motor network synergy. This offers a novel approach and theoretical foundation for integrated cognitive-motor rehabilitation, particularly beneficial for patients exhibiting inadequate cognitive engagement and poor complex task adaptation during recovery.

Several limitations warrant consideration: the relatively small sample size, short intervention duration, and lack of long-term follow-up limited our ability to assess the sustained efficacy of taVNS and the causal relationships among multimodal indicators. The sham protocol, though based on prior taVNS studies, may not have fully matched the sensory experience of active stimulation. Correlation analyses between groups during the intervention were not performed. Stimulation parameters were adopted from published consensus rather than preliminary testing in this cohort. The lesion-specific effects of left-sided taVNS were not evaluated, and although stimulation was synchronized with task execution, it was not precisely matched to discrete movement events. Future studies should include larger samples, explore lesion-specific stimulation protocols, refine sham designs, incorporate intergroup correlation analyses, and optimize stimulation parameters and timing strategies to validate long-term efficacy.

## 6 Conclusion

taVNS paired with TOT promotes post-stroke upper limb functional recovery through synergistic multi-level neuromodulatory mechanisms. These include enhancement of autonomic regulation, elevation of corticomotor pathway excitability, facilitation and activation of cortical regions governing cognitive-motor integration, and reorganization of functional connectivity networks. Crucially, taVNS-induced neural activation patterns and network reconfiguration demonstrate significant cognitive-load-dependent reorganization: During low cognitive demand tasks, activation primarily centers on motor hubs with enhanced local integration, whereas high cognitive demand tasks engage cognitive hubs, driving broader prefrontal-motor network co-activation. This dynamic transition from motor-dominant to cognition-driven processing suggests that taVNS modulates functional coupling within prefrontal-motor cortical pathways according to task cognitive load. Consequently, such neuromodulation optimizes cognitive-motor integration efficiency, augments executive efficiency in complex task performance, and accelerates functional recovery of impaired extremities. Collectively, these findings provide novel neurophysiological evidence supporting individualized rehabilitation strategies for stroke recovery.

## Data Availability

The original contributions presented in the study are included in the article/supplementary material, further inquiries can be directed to the corresponding authors.

## References

[B1] Almhdawi K. A. Jaber H. B. Khalil H. W. Kanaan S. F. Shyyab A. A. Mansour Z. M.. (2021). Post-stroke fatigue level is significantly associated with mental health component of health-related quality of life: a cross-sectional study. Qual. Life Res. 30, 1165–1172. 10.1007/s11136-020-02714-z 33387289

[B2] An S. Oh S. J. Noh S. Jun S. B. Sung J. E. (2025). Enhancing cognitive abilities through transcutaneous auricular vagus nerve stimulation: findings from prefrontal functional connectivity analysis and virtual brain simulation. NeuroImage 311:121179. 10.1016/j.neuroimage.2025.121179 40158670

[B3] Ase H. Honaga K. Tani M. Takakura T. Wada F. Murakami Y.. (2025). Effects of home-based virtual reality upper extremity rehabilitation in persons with chronic stroke: a randomized controlled trial. J. Neuroeng. Rehab. 22:20. 10.1186/s12984-025-01564-5 39901178 PMC11792398

[B4] Bachar Kirshenboim Y. Tzur Lebovich S. Weitzer T. Doron D. Bondi M. Cialic R.. (2025). Upper extremity-cognitive dual-task capacity post-stroke. Neurorehab. Neural Repair 39, 365–376. 10.1177/15459683251317192 39932232 PMC12065953

[B5] Badran B. W. Peng X. Baker-Vogel B. Hutchison S. Finetto P. Rishe K.. (2023). Motor activated auricular vagus nerve stimulation as a potential neuromodulation approach for post-stroke motor rehabilitation: a pilot study. Neurorehab. Neural Repair 37, 374–383. 10.1177/15459683231173357 37209010 PMC10363288

[B6] Balderston N. L. Beer J. C. Seok D. Makhoul W. Deng Z. D. Girelli T.. (2022). Proof of concept study to develop a novel connectivity-based electric-field modelling approach for individualized targeting of transcranial magnetic stimulation treatment. Neuropsychopharmacology 47, 588–598. 10.1038/s41386-021-01110-6 34321597 PMC8674270

[B7] Bestmann S. Krakauer J. W. (2015). The uses and interpretations of the motor-evoked potential for understanding behaviour. Exp. Brain Res. 233, 679–689. 10.1007/s00221-014-4183-7 25563496

[B8] Billinger S. A. Arena R. Bernhardt J. Eng J. J. Franklin B. A. Johnson C. M.. (2014). Physical activity and exercise recommendations for stroke survivors: a statement for healthcare professionals from the American Heart Association/American Stroke Association. Stroke 45, 2532–2553. 10.1161/STR.0000000000000022 24846875

[B9] Camargo L. Pacheco-Barrios K. Gianlorenço A. C. Menacho M. Choi H. Song J. J.. (2024). Evidence of bottom-up homeostatic modulation induced taVNS during emotional and Go/No-Go tasks. Exp. Brain Res. 242, 2069–2081. 10.1007/s00221-024-06876-x 38963558

[B10] De Iaco L. Veerbeek J. M. Ket J. C. F. Kwakkel G. (2024). Upper limb robots for recovery of motor arm function in patients with stroke: a systematic review and meta-analysis. Neurology 103:e209495. 10.1212/WNL.0000000000209495 38870442

[B11] de Melo P. S. Parente J. Rebello-Sanchez I. Marduy A. Gianlorenco A. C. Kyung Kim C.. (2023). Understanding the neuroplastic effects of auricular vagus nerve stimulation in animal models of stroke: a systematic review and meta-analysis. Neurorehab. Neural Repair 37, 564–576. 10.1177/15459683231177595 37272448

[B12] Farmer A. D. Strzelczyk A. Finisguerra A. Gourine A. V. Gharabaghi A. Hasan A.. (2021). International consensus based review and recommendations for minimum reporting standards in research on transcutaneous vagus nerve stimulation (version 2020). Front. Hum. Neurosci. 14:568051. 10.3389/fnhum.2020.568051 33854421 PMC8040977

[B13] Forte G. Favieri F. Leemhuis E. De Martino M. L. Giannini A. M. De Gennaro L.. (2022). Ear your heart: transcutaneous auricular vagus nerve stimulation on heart rate variability in healthy young participants. PeerJ 10:e14447. 10.7717/peerj.14447 36438582 PMC9686410

[B14] Gardi A. Rodriguez K. M. Augenstein T. E. Palmieri-Smith R. M. Krishnan C. (2024). No evidence of hysteresis in quadriceps or hamstring active motor evoked potentials. Restorative Neurol. Neurosci. 42, 231–241. 10.1177/09226028251330850 40239091

[B15] Garrido M. M. Álvarez E E. Acevedo P F. Moyano V Á. Castillo N N. Cavada Ch G. (2023). Early transcranial direct current stimulation with modified constraint-induced movement therapy for motor and functional upper limb recovery in hospitalized patients with stroke: a randomized, multicentre, double-blind, clinical trial. Brain Stimul. 16, 40–47. 10.1016/j.brs.2022.12.008 36584748

[B16] GBD 2021 Diabetes Collaborators (2023). Global, regional, and national burden of diabetes from 1990 to 2021, with projections of prevalence to 2050: a systematic analysis for the Global Burden of Disease Study 2021. Lancet 402, 203–234. 10.1016/S0140-6736(23)01301-6. 37356446 PMC10364581

[B17] Gerges A. N. H. Graetz L. Hillier S. Uy J. Hamilton T. Opie G. Vallence A. M.. (2025). Transcutaneous auricular vagus nerve stimulation modifies cortical excitability in middle-aged and older adults. Psychophysiology 62:e14584. 10.1111/psyp.14584 38602055 PMC11780349

[B18] Gianlorenco A. C. L. de Melo P. S. Marduy A. Kim A. Y. Kim C. K. Choi H.. (2022). Electroencephalographic patterns in taVNS: a systematic review. Biomedicines 10:2208. 10.3390/biomedicines10092208 36140309 PMC9496216

[B19] Giraudier M. Ventura-Bort C. Burger A. M. Claes N. D'Agostini M. Fischer R.. (2022). Evidence for a modulating effect of transcutaneous auricular vagus nerve stimulation (taVNS) on salivary alpha-amylase as indirect noradrenergic marker: a pooled mega-analysis. Brain Stimul. 15, 1378–1388. 10.1016/j.brs.2022.09.009 36183953

[B20] Gondo M. Kawai K. Moriguchi Y. Hiwatashi A. Takakura S. Yoshihara K.. (2023). Effects of integrated hospital treatment on the default mode, salience, and frontal-parietal networks in anorexia nervosa: a longitudinal resting-state functional magnetic resonance imaging study. PLoS ONE 18:e0283318. 10.1371/journal.pone.0283318 37253028 PMC10228763

[B21] Hakim U. De Felice S. Pinti P. Zhang X. Noah J. A. Ono Y.. (2023). Quantification of inter-brain coupling: a review of current methods used in haemodynamic and electrophysiological hyperscanning studies. NeuroImage 280:120354. 10.1016/j.neuroimage.2023.120354 37666393

[B22] Han L. Lu J. Chen C. Ke J. Zhao H. (2023). Altered functional connectivity within and between resting-state networks in patients with vestibular migraine. Neuroradiology 65, 591–598. 10.1007/s00234-022-03102-9 36520172

[B23] Han Z. Zhang C. Cheng K. Chen Y. Tang Z. Chen L.. (2025). Clinical application of respiratory-gated auricular vagal afferent nerve stimulation. Neuroscience 565, 117–123. 10.1016/j.neuroscience.2024.11.065 39615649

[B24] Harrison E. C. Grossen S. Tueth L. E. Haussler A. M. Rawson K. S. Campbell M. C.. (2025). Neural mechanisms underlying synchronization of movement to musical cues in Parkinson disease and aging. Front. Neurosci. 19:1550802. 10.3389/fnins.2025.1550802 40134419 PMC11933100

[B25] Holub F. Petri R. Schiel J. Feige B. Rutter M. K. Tamm S.. (2023). Associations between insomnia symptoms and functional connectivity in the UK Biobank cohort (n = 29,423). J. Sleep Res. 32:e13790. 10.1111/jsr.13790 36528860

[B26] Horinouchi T. Nezu T. Saita K. Date S. Kurumadani H. Maruyama H.. (2024). Transcutaneous auricular vagus nerve stimulation enhances short-latency afferent inhibition via central cholinergic system activation. Sci. Rep. 14:11224. 10.1038/s41598-024-61958-8 38755234 PMC11099104

[B27] Hu Y. Ma J. Chen B. Pang J. Liang W. Wu W. (2024). The duration of chronic pain can affect brain functional changes of the pain matrix in patients with chronic back pain: a resting-state fMRI study. J. Pain Res. 17, 1941–1951. 10.2147/JPR.S457575 38828086 PMC11141710

[B28] Huang Y. Zhang Y. Hodges S. Li H. Yan Z. Liu X.. (2023). The modulation effects of repeated transcutaneous auricular vagus nerve stimulation on the functional connectivity of key brainstem regions along the vagus nerve pathway in migraine patients. Front. Mol. Neurosci. 16:1160006. 10.3389/fnmol.2023.1160006 37333617 PMC10275573

[B29] JAMOVI (2023). Open Statistical Software for the Desktop and Cloud. Available online at: https://www.jamovi.org/ (Accessed June 25, 2025).

[B30] Jonker Z. D. Gaiser C. Tulen J. H. M. Ribbers G. M. Frens M. A. Selles R. W. (2021). No effect of anodal tDCS on motor cortical excitability and no evidence for responders in a large double-blind placebo-controlled trial. Brain Stimul. 14, 100–109. 10.1016/j.brs.2020.11.005 33197654

[B31] Kamran M. A. Mannan M. M. Jeong M. Y. (2016). Cortical signal analysis and advances in functional near-infrared spectroscopy signal: a review. Front. Hum. Neurosci. 10:261. 10.3389/fnhum.2016.00261 27375458 PMC4899446

[B32] Kang D. Choi Y. Lee J. Park E. Kim I. Y. (2024). Analysis of taVNS effects on autonomic and central nervous systems in healthy young adults based on HRV, EEG parameters. J. Neural Eng. 21. 10.1088/1741-2552/ad5d16 38941990

[B33] Keatch C. Lambert E. Woods W. Kameneva T. (2025). Phase-amplitude coupling in response to transcutaneous vagus nerve stimulation: focus on regions implicated in mood and memory. Neuromodulation 28, 663–671. 10.1016/j.neurom.2025.01.011 39998451

[B34] Kwakkel G. van Wegen E. E. H. Burridge J. H. Winstein C. J. van Dokkum L. E. H. Alt Murphy M.. (2019). Standardized measurement of quality of upper limb movement after stroke: consensus-based core recommendations from the second stroke recovery and rehabilitation roundtable. Neurorehab. Neural Repair 33, 951–958. 10.1177/1545968319886477 31660781

[B35] Laborde S. Mosley E. Thayer J. F. (2017). Heart rate variability and cardiac vagal tone in psychophysiological research - recommendations for experiment planning, data analysis, and data reporting. Front. Psychol. 8:213. 10.3389/fpsyg.2017.00213 28265249 PMC5316555

[B36] Lee S. H. Lee G. Kim J. Phillips V Z. Kim H. Kim E.. (2025). Resting-state hemodynamic changes and effects on upper limb function after multi-channel transcranial direct current stimulation to the ipsilesional primary motor cortex and anterior intraparietal sulcus in stroke patients: an fNIRS pilot study. J. Neuroeng. Rehab. 22:83. 10.1186/s12984-025-01618-8 40241110 PMC12001566

[B37] Li C. Song X. Chen S. Wang C. He J. Zhang Y.. (2021a). Long-term effectiveness and adoption of a cellphone augmented reality system on patients with stroke: randomized controlled trial. JMIR Serious Games 9:e30184. 10.2196/30184 34817390 PMC8663710

[B38] Li K. Yang J. Becker B. Li X. (2023). Functional near-infrared spectroscopy neurofeedback of dorsolateral prefrontal cortex enhances human spatial working memory. Neurophotonics 10:025011. 10.1117/1.NPh.10.2.025011 37275655 PMC10234406

[B39] Li R. Zhao C. Wang C. Wang J. Zhang Y. (2020). Enhancing fNIRS analysis using EEG rhythmic signatures: an EEG-informed fNIRS analysis study. IEEE Trans. Bio-Med. Eng. 67, 2789–2797. 10.1109/TBME.2020.2971679 32031925

[B40] Li X. Wu M. Zhang J. Yu D. Wang Y. Su Y.. (2025). Post-stroke dysphagia: neurological regulation and recovery strategies. Biosci. Trends 19, 31–52. 10.5582/bst.2025.01029 39993779

[B41] Li X. Wu Y. Wei M. Guo Y. Yu Z. Wang H.. (2021b). A novel index of functional connectivity: phase lag based on Wilcoxon signed rank test. Cogn. Neurodyn. 15, 621–636. 10.1007/s11571-020-09646-x 34367364 PMC8286916

[B42] Lin S. Rodriguez C. O. Wolf S. L. (2024). Vagus nerve stimulation paired with upper extremity rehabilitation for chronic ischemic stroke: contribution of dosage parameters. Neurorehab. Neural Repair 38, 607–615. 10.1177/15459683241258769 38836606

[B43] Luo Q. Chen J. Li Y. Wu Z. Lin X. Yao J.. (2022). Aberrant brain connectivity is associated with childhood maltreatment in individuals with major depressive disorder. Brain Imaging Behav. 16, 2021–2036. 10.1007/s11682-022-00672-3 35906517

[B44] Machado S. de Oliveira Sant'Ana L. Cid L. Teixeira D. Rodrigues F. Travassos B.. (2022). Impact of victory and defeat on the perceived stress and autonomic regulation of professional eSports athletes. Front. Psychol. 13:987149. 10.3389/fpsyg.2022.987149 36092047 PMC9454608

[B45] Meulenberg C. J. W. Rehfeld K. Jovanović S. Marusic U. (2023). Unleashing the potential of dance: a neuroplasticity-based approach bridging from older adults to Parkinson's disease patients. Front. Aging Neurosci. 15:1188855. 10.3389/fnagi.2023.1188855 37434737 PMC10331838

[B46] Meyers E. C. Solorzano B. R. James J. Ganzer P. D. Lai E. S. Rennaker R. L.. (2018). Vagus nerve stimulation enhances stable plasticity and generalization of stroke recovery. Stroke 49, 710–717. 10.1161/STROKEAHA.117.019202 29371435 PMC6454573

[B47] Owens M. M. Jacquemet V. Napadow V. Lewis N. Beaumont E. (2024). Brainstem neuronal responses to transcutaneous auricular and cervical vagus nerve stimulation in rats. J. Physiol. 602, 4027–4052. 10.1113/JP286680 39031516 PMC11326965

[B48] Paparella G. Rocchi L. Bologna M. Berardelli A. Rothwell J. (2020). Differential effects of motor skill acquisition on the primary motor and sensory cortices in healthy humans. J. Physiol. 598, 4031–4045. 10.1113/JP279966 32639599

[B49] Peng X. Baker-Vogel B. Sarhan M. Short E. B. Zhu W. Liu H.. (2023). Left or right ear? A neuroimaging study using combined taVNS/fMRI to understand the interaction between ear stimulation target and lesion location in chronic stroke. Brain Stimul. 16, 1144–1153. 10.1016/j.brs.2023.07.050 37517466

[B50] Pereira D. J. Pereira J. Sayal A. Morais S. Macedo A. Direito B.. (2024). Functional and structural connectivity success predictors of real-time fMRI neurofeedback targeting DLPFC: Contributions from central executive, salience, and default mode networks. Netw. Neurosci. 8, 81–95. 10.1162/netn_a_00338 38562293 PMC10861170

[B51] Pignolo L. Tonin P. Nicotera P. Bagetta G. Scuteri D. (2022). ROBOCOP (ROBOtic care of poststroke pain): study protocol for a randomized trial to assess robot-assisted functional and motor recovery and impact on poststroke pain development. Front. Neurol. 13:813282. 10.3389/fneur.2022.813282 35250820 PMC8894665

[B52] Potts C. A. Williamson R. A. Jacob J. D. Kantak S. S. Buxbaum L. J. (2024). Reaching the cognitive-motor interface: effects of cognitive load on arm choice and motor performance after stroke. Exp. Brain Res. 242, 2785–2797. 10.1007/s00221-024-06939-z 39395059 PMC11869378

[B53] Rajda C. M. Desabrais K. Levin M. F. (2025). Relationships between cognitive impairments and motor learning after stroke: a scoping review. Neurorehab. Neural Repair 39, 142–156. 10.1177/15459683241300458 39606925 PMC11849258

[B54] Rodrigues D. F. Neves V. R. Montarroyos U. R. Dos Santos W. J. de Farias I. C. V. Filho D. C. S. (2024). Association of heart rate variability with cardiorespiratory fitness and muscle strength in patients after hospitalization for COVID-19: an analytical cross-sectional study. Clinics 79:100534. 10.1016/j.clinsp.2024.100534 39566371 PMC11617900

[B55] Shi X. Zhao J. Xu S. Ren M. Wu Y. Chen X.. (2023). Clinical research progress of the post-stroke upper limb motor function improvement via transcutaneous auricular vagus nerve stimulation. Neural Plasticity 2023:9532713. 10.1155/2023/9532713 37789954 PMC10545466

[B56] Sintini I. Corriveau-Lecavalier N. Jones D. T. Machulda M. M. Gunter J. L. Schwarz C. G.. (2024). Longitudinal default mode sub-networks in the language and visual variants of Alzheimer's disease. Brain Commun. 6:fcae005. 10.1093/braincomms/fcae005 38444909 PMC10914456

[B57] Sommer A. Fischer R. Borges U. Laborde S. Achtzehn S. Liepelt R. (2023). The effect of transcutaneous auricular vagus nerve stimulation (taVNS) on cognitive control in multitasking. Neuropsychologia 187:108614. 10.1016/j.neuropsychologia.2023.108614 37295553

[B58] Steidel K. Krause K. Menzler K. Strzelczyk A. Immisch I. Fuest S.. (2021). Transcutaneous auricular vagus nerve stimulation influences gastric motility: a randomized, double-blind trial in healthy individuals. Brain Stimul. 14, 1126–1132. 10.1016/j.brs.2021.06.006 34187756

[B59] Szeska C. Klepzig K. Hamm A. O. Weymar M. (2025). Ready for translation: non-invasive auricular vagus nerve stimulation inhibits psychophysiological indices of stimulus-specific fear and facilitates responding to repeated exposure in phobic individuals. Transl. Psychiatry 15:135. 10.1038/s41398-025-03352-0 40204704 PMC11982236

[B60] van Midden V. M. Demšar J. Pirtošek Z. Kojović M. (2023). The effects of transcutaneous auricular vagal nerve stimulation on cortical GABAergic and cholinergic circuits: a transcranial magnetic stimulation study. Euro. J. Neurosci. 57, 2160–2173. 10.1111/ejn.16004 37125748

[B61] Viglione A. Mazziotti R. Pizzorusso T. (2023). From pupil to the brain: new insights for studying cortical plasticity through pupillometry. Front. Neural Circuits 17:1151847. 10.3389/fncir.2023.1151847 37063384 PMC10102476

[B62] Wang M. H. Jin Y. J. He M. F. Zhou A. N. Zhu M. L. Lin F.. (2024a). Transcutaneous auricular vagus nerve stimulation improves cognitive decline by alleviating intradialytic cerebral hypoxia in hemodialysis patients: a fNIRS pilot study. Heliyon 10:e39841. 10.1016/j.heliyon.2024.e39841 39975458 PMC11838084

[B63] Wang M. H. Wang Y. X. Xie M. Chen L. Y. He M. F. Lin F.. (2024b). Transcutaneous auricular vagus nerve stimulation with task-oriented training improves upper extremity function in patients with subacute stroke: a randomized clinical trial. Front. Neurosci. 18:1346634. 10.3389/fnins.2024.1346634 38525376 PMC10957639

[B64] Wang X. Yin L. Wang Y. Zhang H. Zhang S. Wu J.. (2024c). Transcutaneous electrical acupoint stimulation for upper limb motor recovery after stroke: a systematic review and meta-analysis. Front. Aging Neurosci. 16:1438994. 10.3389/fnagi.2024.1438994 39665041 PMC11631906

[B65] Wang Y. Zhang J. Li Y. Qi S. Zhang F. Ball L. J.. (2023). Preventing prefrontal dysfunction by tDCS modulates stress-induced creativity impairment in women: an fNIRS study. Cereb. Cortex 33, 10528–10545. 10.1093/cercor/bhad301 37585735

[B66] Wei D. Hua X. Y. Zheng M. X. Wu J. J. Xu J. G. (2022). Effectiveness of robot-assisted virtual reality mirror therapy for upper limb motor dysfunction after stroke: study protocol for a single-center randomized controlled clinical trial. BMC Neurol. 22:307. 10.1186/s12883-022-02836-6 35996106 PMC9396805

[B67] Wheelock M. D. Strain J. F. Mansfield P. Tu J. C. Tanenbaum A. Preische O.. (2023). Brain network decoupling with increased serum neurofilament and reduced cognitive function in Alzheimer's disease. Brain 146, 2928–2943. 10.1093/brain/awac498 36625756 PMC10316768

[B68] Wu Y. Tao C. Li Q. (2024). Fatigue characterization of EEG brain networks under mixed reality stereo vision. Brain Sci. 14:1126. 10.3390/brainsci14111126 39595889 PMC11591834

[B69] Yokota H. Edama M. Hirabayashi R. Sekine C. Otsuru N. Saito K.. (2022). Effects of stimulus frequency, intensity, and sex on the autonomic response to transcutaneous vagus nerve stimulation. Brain Sci. 12:1038. 10.3390/brainsci12081038 36009101 PMC9405815

[B70] Yun Y. J. Myong Y. Oh B. M. Song J. J. Kim C. K. Seo H. G. (2025). Effects of transcutaneous auricular vagus nerve stimulation on cortical excitability in healthy adults. Neuromodulation 28, 115–122. 10.1016/j.neurom.2024.05.004 38878053

[B71] Zhou S. Huang Y. Jiao J. Hu J. Hsing C. Lai Z.. (2021). Impairments of cortico-cortical connectivity in fine tactile sensation after stroke. J. Neuroeng. Rehab. 18:34. 10.1186/s12984-021-00821-7 33588877 PMC7885375

[B72] Zhuang Y. Zhai W. Li Q. Jiao H. Ge Q. Rong P.. (2023). Effects of simultaneous transcutaneous auricular vagus nerve stimulation and high-definition transcranial direct current stimulation on disorders of consciousness: a study protocol. Front. Neurol. 14:1165145. 10.3389/fneur.2023.1165145 37693756 PMC10483839

[B73] Zou N. Zhou Q. Zhang Y. Xin C. Wang Y. Claire-Marie R.. (2024). Transcutaneous auricular vagus nerve stimulation as a novel therapy connecting the central and peripheral systems: a review. Int. J. Surg. 110, 4993–5006. 10.1097/JS9.0000000000001592 38729100 PMC11326027

